# APOE genotype and the effect of statins on lipid outcomes: A meta‐analysis

**DOI:** 10.1002/bcp.70493

**Published:** 2026-02-17

**Authors:** Innocent G. Asiimwe, Tsegay Gebru, Andrea L. Jorgensen, Munir Pirmohamed, Multimorbidity Mechanism and Therapeutics Research Collaborative

**Affiliations:** ^1^ Department of Pharmacology and Therapeutics, Institute of Systems, Molecular and Integrative Biology University of Liverpool Liverpool UK; ^2^ Department of Health Data Science, Institute of Population Health Sciences University of Liverpool Liverpool UK

**Keywords:** *APOE* genotype, lipid response, meta‐analysis, statins

## Abstract

**Aim:**

*APOE* genotype may affect statin therapy response. We conducted a meta‐analysis to update and quantify this association across various outcomes.

**Methods:**

We searched seven databases (MEDLINE, Scopus, Web of Science, the Cochrane Library, APA PsycINFO, CINAHL Plus and ClinicalTrials.gov) on 9 May 2024. Screening and data extraction were performed by two reviewers and a machine learning tool (ASReview).

**Results:**

From 4352 de‐duplicated records, 52 studies were included in the meta‐analysis. Biomarkers analysed included low‐density lipoprotein cholesterol (LDLC), total cholesterol (TC), triglycerides (TG) and high‐density lipoprotein cholesterol (HDLC). Compared to *ε3* carriers, *ε2* carriers showed greater reductions in LDLC in response to statin treatment (mean difference in percentage change: −2.98%, 95% CI: −5.88% to −0.08%) and similar reductions in TC (−2.73%, −5.62% to 0.16%), and TG (−4.95%, −11.93% to 2.04%) with no significant difference in HDLC (−0.09%, −3.10% to 2.91%). After adjusting for publication bias, *ε4* carriers showed less pronounced statin effects, with smaller reductions in LDLC (mean difference: 10.04%, 6.04% to 14.04%), TC (8.99%, 5.08% to 12.90%) and TG (8.24%, 2.15% to 14.33%), along with a smaller increase in HDLC (−10.08%, −15.30% to −4.85%) compared to *ε3* carriers. Study quality was unclear, and heterogeneity (partly explained by sex and Familial hypercholesterolemia) was high, especially for the percentage changes. A stronger genotype effect was seen in males.

**Conclusion:**

Our meta‐analysis shows that *APOE* genotype may influence statin response, emphasizing the need to incorporate known genetic factors into personalized treatment regimens.

## 1 INTRODUCTION

Cardiovascular diseases (CVD) remain the leading cause of death worldwide. In 2021, despite the COVID‐19 pandemic, ischaemic heart disease was the leading cause of age‐standardized deaths globally, with 108.7 deaths (95% uncertainty interval [UI] 99.8–115.6) per 100 000 population.^1^ Stroke, overtaken by COVID‐19, ranked third with 87.4 deaths (95% UI 79.5–94.4) per 100 000 population.^1^ Randomized controlled trials (RCTs) have consistently demonstrated the efficacy of statins, or 3‐hydroxymethyl‐3‐methylglutaryl coenzyme A (HMG‐CoA) reductase inhibitors, in reducing overall mortality, establishing them as standard treatments for primary and secondary prevention of cardiovascular disease. For example, a 2022 systematic review evaluating statin use for the primary prevention of CVD reported that statins significantly reduced the risk of all‐cause mortality (risk ratio [RR] 0.92 [95% confidence interval/CI, 0.87–0.98]).^2^ This review included evidence from 22 RCTs with a total of 90 624 participants. Statins were also protective against CVD, reducing the risk of fatal or nonfatal stroke (RR 0.78 [95% CI 0.68–0.90]) and myocardial infarction (RR 0.67 [95% CI 0.60–0.75]).^2^

The Apolipoprotein E (*APOE*) gene, located on the long arm of chromosome 19 (19q13.32), is both a CVD risk factor and a modulator of statin therapy.^3^, ^4^, ^5^ It encodes the Apolipoprotein E (ApoE) protein, which plays a crucial role in lipid metabolism and is present in triglyceride‐rich chylomicrons, very‐low‐density lipoproteins (VLDL), intermediate‐density lipoproteins (IDL) and certain high‐density lipoprotein (HDL) subclasses. There are three primary isoforms of ApoE (*ε2*, *ε3* and *ε4*) resulting from two single‐nucleotide polymorphisms (SNPs), rs429358 (T > C) and rs7412 (C > T). These SNPs result in key amino acid changes; specifically, rs429358 causes a cysteine (amino acid codon TGC) to arginine (CGC) change at position 112, and rs7412 causes an arginine to cysteine change at position 158. The *ε2* isoform has cysteine residues at both positions 112 and 158, the *ε3* isoform has cysteine at 112 and arginine at 158 and the *ε4* isoform has arginine at both positions.^6^, ^7^, ^8^ The *ε3* allele is the most common, found in over 60% of the population.^4^, ^7^ These three alleles can form six genotypes including three homozygotes (*ε2ε2*, *ε3ε3* and *ε4ε4*) and three heterozygotes (*ε2ε3*, *ε2ε4* and *ε3ε4*).

ApoE isoforms affect the metabolism and clearance of lipoproteins through interactions with receptors such as the low‐density lipoprotein (LDL) receptor (LDLR).^6^, ^8^, ^9^ The *ε2* isoform has defective binding to LDLR leading to delayed clearance, higher plasma ApoE levels and upregulation of HMG‐CoA and LDLR synthesis, resulting in lower plasma total cholesterol (TC) and LDL cholesterol (LDLC) levels. Conversely, the *ε4* isoform is cleared more rapidly, causing downregulation of HMG‐CoA and LDLR and consequently higher plasma TC and LDLC levels. These genotype‐specific differences in baseline lipid metabolism provide a biological rationale for potential variation in statin responsiveness. Supporting this, Bennet et al.^10^ analysed lipid profiles from 82 studies (86 067 healthy participants) and demonstrated a consistent gradient in LDLC levels across *APOE* genotypes, with *ε2* carriers showing the lowest and *ε4* carriers the highest levels. Although *ε2* is generally associated with a more favourable lipid profile, individuals homozygous for *ε2* allele may rarely develop familial dysbetalipoproteinemia, a condition characterized by markedly abnormal lipid metabolism and increased cardiovascular risk.^4^, ^11^

As reviewed by Nieminen et al.,^8^ *APOE* genotype can influence responses to statin therapy, with *ε2* carriers generally experiencing greater reductions in LDLC levels compared to *ε4* carriers. However, if baseline LDLC levels are substantially higher in *ε4* carriers, they may show larger percentage reductions in response to statin therapy when compared with *ε2* or *ε3* carriers who start with lower baseline levels.^12^ Understanding the impact of *APOE* genotype on lipid metabolism and statin efficacy is crucial for managing cardiovascular risk and tailoring treatment strategies.

Previous studies examining the role of *APOE* genotype in statin response have produced inconsistent findings. For example, a 2009 systematic review analysed 24 studies and found no significant differences in the pooled mean reduction of total cholesterol among the genotypes: *ε2* carriers had a reduction of −27.7% (95% CI: −32.5 to −22.8%), *ε3ε3* had −25.3% (95% CI: −28.0 to −22.6%) and *ε4* carriers had −25.1% (95% CI: −29.3 to −21.0%), corresponding to mean differences of −2.4% between *ε2* and *ε3* carriers and 0.2% between *ε4* and *ε3* carriers.^13^ Similarly, there were no significant differences in LDLC, HDL cholesterol or triglyceride levels across the genotype groups. However, a 2014 pharmacogenetic meta‐analysis of genome‐wide association studies confirmed a role for *APOE* (specifically the rs445925 SNP) LDLC response to statin therapy.^14^

Because of these discrepancies, the existing evidence is still considered insufficient; the Clinical Pharmacogenetics Implementation Consortium currently deems the data inadequate to support clinical guidelines for *APOE*–statin interactions (https://cpicpgx.org/genes-drugs/). Building on our recent work,^15^ which used large datasets with linked electronic health records to examine relationships between *APOE* genotype, statin use and outcomes such as lipid level changes and all‐cause mortality, this meta‐analysis aimed to further strengthen the evidence base by quantifying the association between *APOE* genotype and responses to statin therapy. Given the limited reporting of clinical events, lipid outcomes were selected as the primary endpoints.

## 2 METHODS

This study followed a predefined protocol (PROSPERO: CRD42024545603) and is reported as per the Preferred Reporting Items for Systematic Reviews and Meta‐Analyses (PRISMA) 2020^16^ and Meta‐analysis Of Observational Studies in Epidemiology (MOOSE)^17^ statements (Table S1).

### 2.1 Search strategy and selection criteria

We searched seven databases (MEDLINE, Scopus, Web of Science, the Cochrane Library, APA PsycInfo, CINAHL Plus and ClinicalTrials.gov) on 9 May 2024 using medical subject headings and keywords related to *APOE* and ‘statins’ (detailed search strategies are provided in Table S2). Additionally, we manually searched reference lists from identified studies and previous systematic reviews, and we contacted experts to identify further eligible articles.

We included all studies regardless of their publication year or status. Both observational studies (e.g., retrospective or prospective cohort and case–control studies) and interventional studies (e.g., randomized controlled trials) were considered if they investigated the association between (a) Apolipoprotein E (*APOE*) genotype (*APOE* SNPs such as rs429358 and rs7412 and *APOE* carrier status), (b) statins (including atorvastatin, cerivastatin, fluvastatin, lovastatin, pitavastatin, pravastatin and simvastatin) and (c) any clinical outcomes related to safety and efficacy, including lipid levels (primary endpoints), reported in the primary papers. We excluded case reports, review articles, letters, commentaries and editorials unless they contained information on primary studies not published elsewhere. Non‐English studies without translation and any studies from which data could not be extracted were also excluded.

### 2.2 Data extraction and quality assessment

The screening of titles, abstracts and full texts of all retrieved bibliographic records was conducted by two reviewers: IGA (who screened all records) and TG (who screened MEDLINE records), along with a machine learning framework, ASReview.^18^ One reviewer (IGA) repeated screening using ASReview, with stopping criteria based on the number of records selected by the human reviewers. For example, if human reviewers selected 100 records during abstract screening, ASReview‐assisted screening was stopped once ASReview had identified 100 records. If an abstract was unavailable, the full text was obtained unless the article could be confidently excluded based on its title alone. In cases of uncertainty regarding a study's eligibility, it proceeded to the full text screening stage to minimize the risk of erroneously excluding relevant studies.

We created and piloted a data extraction form using a randomly selected subset of included papers to capture pertinent details such as study design, patient characteristics, study quality and outcomes. When multiple studies analysed the same dataset (identified based on study acronyms, recruitment sites and periods, and authors and their affiliations) for a specific exposure‐outcome combination, we prioritized peer‐reviewed publications and those reporting data from a larger number of patients to avoid duplicate participant inclusion. We used WebPlotDigitizer (Version 4, https://apps.automeris.io/wpd4/) to digitize and extract data (both central tendency and variability measures) presented only in figures.

To assess the methodological quality of each included study, we planned to use the checklist provided by the STrengthening the Reporting Of Pharmacogenetic Studies (STROPS) guideline.^19^

### 2.3 Data synthesis and analysis

When two or more studies were available for variant‐outcome combinations, we obtained pooled estimates by conducting pairwise meta‐analyses comparing heterozygotes *vs*. wild‐type homozygotes, mutant‐type homozygotes *vs*. wild‐type homozygotes and/or mutant‐type homozygotes plus heterozygotes *vs*. wild‐type homozygotes. For *APOE* carrier status, we conducted the following analyses: *ε2* carriers *vs. ε3* carriers, *ε4* carriers *vs. ε3* carriers, *ε2* carriers *vs. ε2* noncarriers and *ε4* carriers *vs. ε4* noncarriers. In this context, *ε2* carriers included *ε2ε2* and *ε2ε3*, *ε3* carriers included *ε3ε3*, and *ε4* carriers included *ε3ε4* and *ε4ε4*. Due to the opposing effects of the *ε2* and *ε4* alleles, individuals with the *ε2ε4* genotype were initially excluded. However, sensitivity analyses were conducted, including *ε2ε4* individuals classified as *ε2*, *ε3* or *ε4* carriers.

We considered outcomes recorded at baseline, posttreatment or as changes from baseline separately. In each analysis, we ensured uniform units and used conversion factors reported in the literature. For example, to convert total cholesterol, low‐density lipoprotein cholesterol and high‐density lipoprotein cholesterol from mg/dL to mmol/L, we divided the value in mg/dL by 38.67. To convert Apolipoprotein B from mg/dL to mmol/L, we assumed that 0.0512 mg/dL equals 1 000 000 mmol/L.^20^

Meta‐analyses were performed using the meta^21^ package in R (Version 4.4.0). We generated odds ratios for dichotomous outcomes and mean differences for continuous outcomes, together with corresponding 95% confidence intervals. For studies reporting odds ratios, we pooled the reported estimates, giving priority to the estimates adjusting for the most covariates when multiple estimates were available. For continuous outcomes, we estimated means and standard deviations from provided median values and interquartile ranges^22^, ^23^ and combined means and standard deviations using formulas from the Cochrane Handbook^23^ (Table S3) when necessary. Forest plots were created for the exposure‐outcome combinations to visually represent the results.

We evaluated the magnitude of inconsistency in study results through visual inspection of forest plots and by considering the *I* ^2^ statistic,^23^ categorizing heterogeneity as low (*I* ^2^ < 30%), moderate (30–70%) or high (>70%). Potential sources of heterogeneity were explored in subgroup analyses based on factors such as sex and Familial Hypercholesterolemia.

When 10 or more studies were available for a given exposure‐outcome combination, we assessed publication bias using the linear regression test of funnel plot asymmetry. A *p* value of <0.1 was considered indicative of publication bias. If visual assessment suggested publication bias, we conducted exploratory analyses and adjusted for it using trim and fill analysis.

### 2.4 Nomenclature of targets and ligands

Key protein targets and ligands in this article are hyperlinked to corresponding entries in http://www.guidetopharmacology.org and are permanently archived in the Concise Guide to PHARMACOLOGY 2023/24.^97^

## 3 RESULTS

**FIGURE 1:**
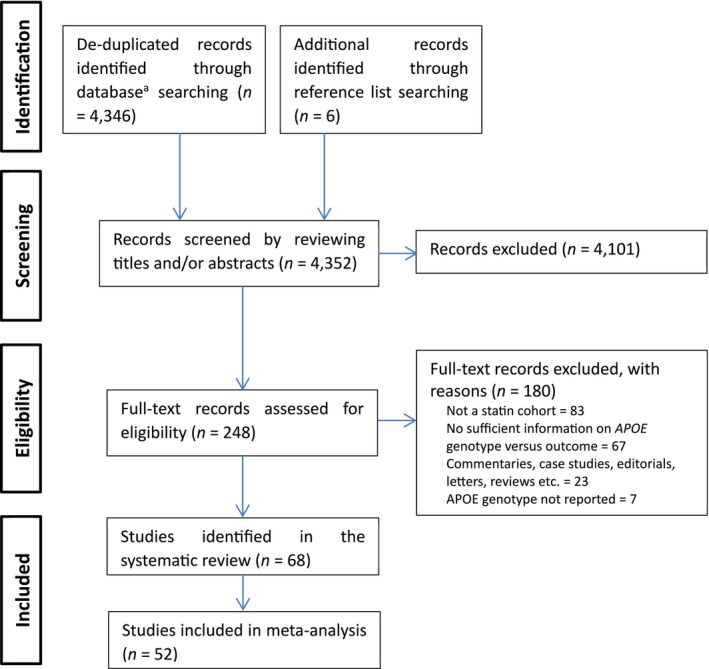
Flow chart of included studies. ^a^Databases included MEDLINE, Scopus, web of science, the Cochrane library, APA PsycInfo, CINAHL plus, and ClinicalTrials.Gov. *APOE*, apolipoprotein E.

We conducted a systematic review to identify studies for inclusion in the meta‐analysis. Figure 1 shows the literature search and selection process. From 4352 unique records identified, 68 were identified in the systematic review. The characteristics of these studies are detailed in Table S4, whereas Tables S5–S7 present the extracted results for the ratio, binary and continuous outcomes, respectively. The median publication year of these studies was 2008, with an interquartile range from 2002 to 2018.

We initially planned to assess the methodological quality of the included studies using the STROPS checklist. However, given that more than half of these studies (56%, or 38 out of 68) were published before 2010 (with 15%, or 10 out of 68, published before 2000), many key items (such as the Reference SNP cluster ID, sample size, genotype quality control methods and population stratification) were not mandatory at that time. Based on a randomly selected subset of included papers, and due to the frequent lack of required information, we decided not to assess the methodological quality.

### 3.1 Studies and outcomes included in the meta‐analysis

**FIGURE 2:**
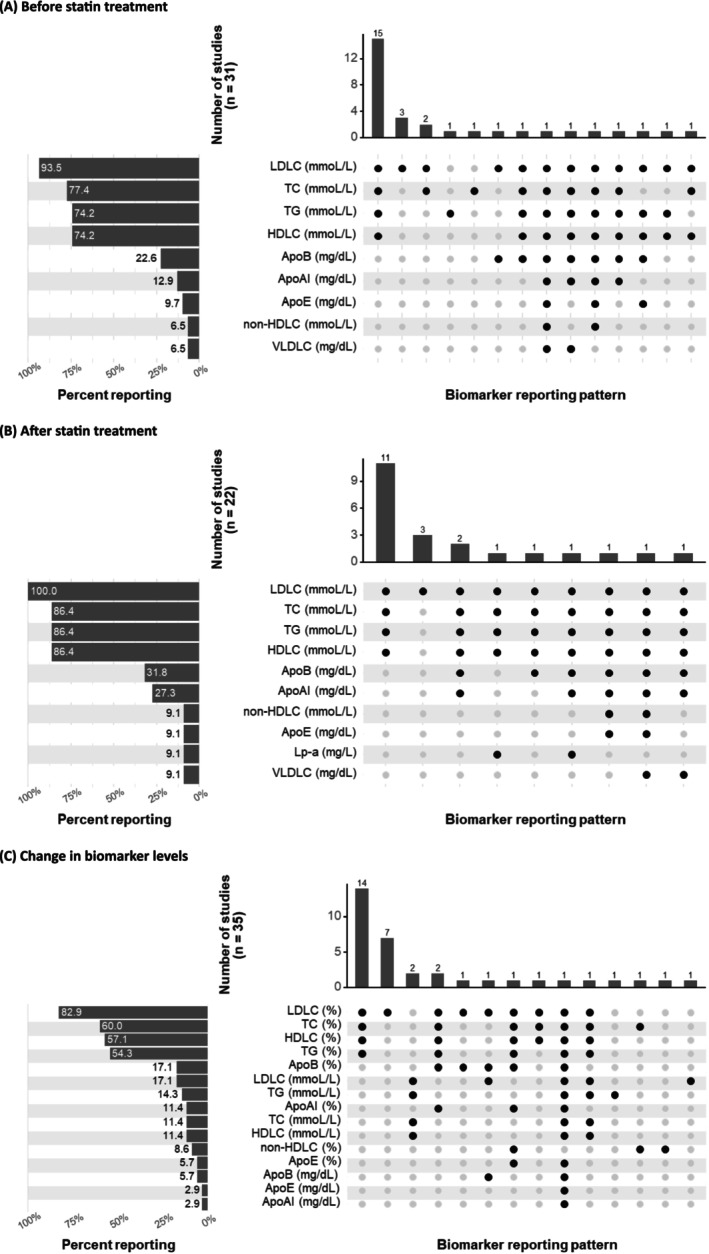
Studies included in the quantitative synthesis (meta‐analysis) for the continuous biomarkers. (A) Studies reporting biomarker measurement before statin treatment. (B) Studies reporting biomarker measurement after statin treatment. (C) Studies reporting change in biomarker levels. The left panels show the frequency with which biomarkers were reported. For example, in panel A, LDLC (analysed in mmol/L) was the most frequently reported biomarker, appearing in 93.5% (29 out of 31) of the studies that measured biomarkers before statin treatment. On the other hand, the right panels show the reporting patterns. For example, in panel A, the most common combination of reported outcomes (first column) included LDLC, TC, TG and HDLC (all analysed in mmol/L), which were reported together in 15 studies. ApoAI, apolipoprotein AI; ApoB, apolipoprotein B; ApoE, apolipoprotein E; HDLC, high‐density lipoprotein cholesterol; LDLC, low‐density lipoprotein cholesterol; Lp‐a, lipoprotein(a); TC, total cholesterol; TG, total triglycerides; VLDLC, very low‐density lipoprotein cholesterol.

Of the 68 studies identified in the systematic review,^3^, ^12^, ^24^, ^25^, ^26^, ^27^, ^28^, ^29^, ^30^, ^31^, ^32^, ^33^, ^34^, ^35^, ^36^, ^37^, ^38^, ^39^, ^40^, ^41^, ^42^, ^43^, ^44^, ^45^, ^46^, ^47^, ^48^, ^49^, ^50^, ^51^, ^52^, ^53^, ^54^, ^55^, ^56^, ^57^, ^58^, ^59^, ^60^, ^61^, ^62^, ^63^, ^64^, ^65^, ^66^, ^67^, ^68^, ^69^, ^70^, ^71^, ^72^, ^73^, ^74^, ^75^, ^76^, ^77^, ^78^, ^79^, ^80^, ^81^, ^82^, ^83^, ^84^, ^85^, ^86^, ^87^, ^88^, ^89^ 52 were included in the meta‐analysis.^3^, ^12^, ^24^, ^25^, ^26^, ^27^, ^28^, ^29^, ^30^, ^31^, ^32^, ^33^, ^34^, ^35^, ^36^, ^37^, ^38^, ^39^, ^40^, ^41^, ^42^, ^43^, ^44^, ^45^, ^46^, ^47^, ^48^, ^49^, ^50^, ^51^, ^52^, ^53^, ^54^, ^55^, ^56^, ^57^, ^58^, ^59^, ^60^, ^61^, ^62^, ^63^, ^64^, ^65^, ^66^, ^67^, ^68^, ^69^, ^70^, ^71^, ^72^, ^73^ This included one study^24^ that reported adjusted odds ratios for two cohorts (risk of lobar intracranial haemorrhage outcome), two studies^25^, ^26^ that reported mortality as a binary outcome and 49 studies^3^, ^12^, ^27^, ^28^, ^29^, ^30^, ^31^, ^32^, ^33^, ^34^, ^35^, ^36^, ^37^, ^38^, ^39^, ^40^, ^41^, ^42^, ^43^, ^44^, ^45^, ^46^, ^47^, ^48^, ^49^, ^50^, ^51^, ^52^, ^53^, ^54^, ^55^, ^56^, ^57^, ^58^, ^59^, ^60^, ^61^, ^62^, ^63^, ^64^, ^65^, ^66^, ^67^, ^68^, ^69^, ^70^, ^71^, ^72^, ^73^ that reported continuous outcomes (Figure 2). Figure 2 is stratified by the time of biomarker measurement and includes 31 studies that reported biomarker measurements before statin treatment, 22 studies that reported measurements after statin treatment and 35 studies that reported changes in biomarker levels.

Across all time points, low‐density lipoprotein cholesterol (LDLC) was the most frequently reported continuous biomarker, included in 93.5% of the 31 studies before statin treatment, 100% of the 22 studies after treatment and 82.9% of the 35 studies reporting biomarker changes (both net/actual and percentage changes). Total cholesterol (TC) was reported in 77.4% of studies before statin treatment, 86.4% after treatment and 60.0% of studies reporting biomarker changes. Total triglycerides (TGs) were included in 74.2% of studies before statin treatment, 86.4% after and 54.3% of studies reporting changes. High‐density lipoprotein cholesterol (HDLC) was reported in 74.2% of studies before treatment, 86.4% after and 57.1% of studies reporting changes. Six other biomarkers were reported in at least two studies but less than a third of studies: Apolipoprotein AI (ApoAI), Apolipoprotein B (ApoB), Apolipoprotein E (ApoE), Lipoprotein(a) [Lp(a)], non‐HDLC and very low‐density lipoprotein cholesterol (VLDLC), as shown in Figure 2.

### 3.2 Meta‐analysis results

#### 3.2.1 Low‐density lipoprotein cholesterol (LDLC)

**FIGURE 3:**
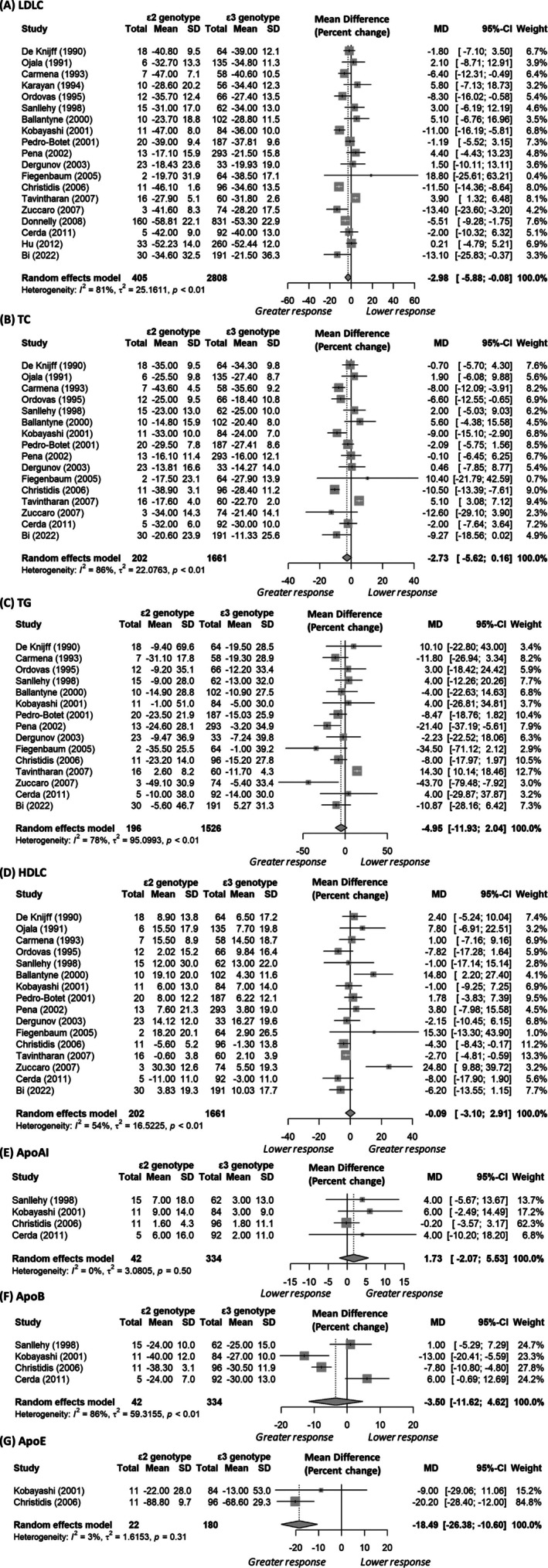
Forest plots comparing apolipoprotein *ε2* carriers with *ε3* carriers, excluding individuals with the *ε2ε4* genotype. For all biomarkers except HDLC and ApoAI, values greater than zero indicate a lower response to statin treatment in *ε2* carriers compared to *ε3* carriers (controls). Abbreviations: ApoAI, apolipoprotein AI; ApoB, apolipoprotein B; ApoE, apolipoprotein E; HDLC, high‐density lipoprotein cholesterol; LDLC, low‐density lipoprotein cholesterol; TC, total cholesterol; TG, total triglycerides.

Due to the opposing effects of the *ε2* and *ε4* alleles, individuals with the *ε2ε4* genotype were initially excluded from the analysis. Significant associations between LDLC levels and *APOE* were found for the *ε2 vs. ε3* (control) genotype both before (22 studies, 4029 participants; mean difference: −0.31 mmol/L, 95% CI: −0.53 to −0.08, *I* ^2^ = 71%) and after statin treatment (17 studies, 2275 participants; −0.41 mmol/L, 95% CI: −0.68 to −0.14, *I* ^2^ = 88%, Tables 1 and S8). For the percentage change in biomarkers before to after statin treatment, *ε2* carriers showed a more pronounced statin effect, with greater reductions in LDLC compared to *ε3* carriers (19 studies, 3213 participants; mean difference: −2.98%, 95% CI: −5.88% to −0.08%), although heterogeneity was high (*I* ^*2*^ = 81%, Figure 3A). A sex‐stratified analysis partially accounted for this heterogeneity, with the male‐specific analysis remaining significant (6 studies, 1025 participants; mean difference: −5.07%, 95% CI: −7.92% to −2.22%, *I* ^2^ = 20%). The analysis of the net/actual change in LDLC levels before and after statin treatment showed a similar pattern to the percentage change, with *ε2* carriers having a greater, although not statistically significant, LDLC reduction (5 studies, 874 participants; mean difference: −0.14 mmol/L, 95% CI ‐0.28 to 0.003, *I* ^2^ = 42%; Table 1 and Figure S1).

**TABLE 1:** Meta‐analysis results with the *ε2ε4* genotype category excluded.

		Before statin treatment			After statin treatment			Change in biomarker			Percentage change in biomarker									
											Main results			Trim‐fill analysis^a^			Subgroup analysis			
Outcome (units)	Analysis	*n*	*N*	Pooled estimates^b^	*n*	*N*	Pooled estimates^b^	*n*	*N*	Pooled estimates^a^	*n*	*N*	Pooled estimates^b^	*n*	*N*	Pooled estimates^b^	Subgroup	*n*	*N*	Pooled estimates^b^
LDLC (mmol/L)	*ε2 vs. ε3*	22	4029	***−0.31 (−0.53; −0.08), I*** ^***2***^ ***= 71%***	17	2275	***−0.41 (−0.68; −0.14), I*** ^***2***^ ***= 88%***	5	874	−0.14 (−0.28; 0.003), *I* ^*2*^ = 42%	19	3213	***−2.98% (−5.88; −0.08), I*** ^***2***^ ***= 81%***	NA	NA	NA	Female	6	847	−2.84% (−8.81; 3.14), *I* ^*2*^ = 71%
																	Male	6	1025	***−5.07% (−7.92; −2.22), I*** ^***2***^ ***= 20%***
	*ε4 vs. ε3*	26	6275	0.01 (−0.09; 0.11), *I* ^*2*=^68%	21	4178	0.08 (−0.10; 0.25), *I* ^*2*^ = 96%	5	1032	***0.30 (0.06; 0.53), I*** ^***2***^ ***= 74%***	21	4926	***2.91% (0.18; 5.65), I*** ^***2***^ ***= 97%***	32	6326	***10.04% (6.04; 14.04), I*** ^***2***^ ***= 98%***	Female	6	1053	***3.40% (0.50; 6.29), I*** ^***2***^ ***= 44%***
																	Male	6	1235	***3.91% (1.55; 6.27), I*** ^***2***^ ***= 22%***
																	Non‐FH	2	323	2.52% (−0.51; 5.55), *I* ^*2*^ = 0%
																	FH	2	530	***2.51% (0.24; 4.77), I*** ^***2***^ ***= 0%***
TC (mmol/L)	*ε2 vs. ε3*	17	1555	−0.13 (−0.38; 0.13), *I* ^*2*^ = 81%	15	1161	***−0.32 (−0.62; −0.01), I*** ^***2***^ ***= 83%***	4	628	−0.22 (−0.49; 0.05), *I* ^*2*^ = 49%	16	1863	−2.73% (−5.62; 0.16), *I* ^*2*^ = 86%	NA	NA	NA	Female	4	338	−1.63% (−7.96; 4.71), *I* ^*2*^ = 51%
																	Male	4	461	***−6.27% (−9.12; −3.41), I*** ^***2***^ ***= 3%***
	*ε4 vs. ε3*	22	3419	−0.04 (−0.17; 0.10), *I* ^*2*^ = 79%	19	2869	0.11 (−0.05; 0.28), *I* ^*2*^ = 92%	4	815	***0.20 (0.05; 0.34), I*** ^***2***^ ***= 38%***	18	3305	2.11% (−0.36; 4.58), *I* ^*2*^ = 97%	28	4583	***8.99% (5.08; 12.90), I*** ^***2***^ ***= 98%***	Female	4	437	1.32% (−0.69; 3.34), *I* ^*2*^ = 0%
																	Male	4	564	4.15% (−0.34; 8.64), *I* ^*2*^ = 73%
TG (mmol/L)	*ε2 vs. ε3*	17	2508	***0.28 (0.004; 0.56), I*** ^***2***^ ***= 75%***	13	1156	***0.26 (0.11; 0.41), I*** ^***2***^ ***= 23%***	5	661	−0.17 (−0.33; 0.0003), *I* ^*2*^ = 0%	15	1722	−4.95% (−11.93; 2.04), *I* ^*2*^ = 78%	23	2883	10.41% (−0.19; 21.00), *I* ^*2*^ = 87%	Female	4	338	−14.48% (−29.03; 0.07), *I* ^*2*^ = 66%
																	Male	4	461	***−9.59% (−19.18; −0.01), I*** ^***2***^ ***= 0%***
	*ε4 vs. ε3*	21	4248	−0.01 (−0.07; 0.05), *I* ^*2*^ = 47%	17	2678	0.05 (−0.05; 0.15), *I* ^*2*^ = 83%	5	851	−0.04 (−0.12; 0.04), *I* ^*2*^ = 0%	17	3087	−0.78% (−5.06; 3.49), *I* ^*2*^ = 86%	26	4265	***8.24% (2.15; 14.33), I*** ^***2***^ ***= 88%***	Female	4	437	−3.81% (−9.80; 2.17), *I* ^*2*^ = 0%
																	Male	4	564	−2.40% (−11.91; 7.11), *I* ^*2*^ = 56%
HDLC (mmol/L)	*ε2 vs. ε3*	17	2616	***−0.07 (−0.13; −0.01), I*** ^***2***^ ***= 73%***	13	1156	***−0.09 (−0.15; −0.02), I*** ^***2***^ ***= 33%***	5	628	***0.04 (0.004; 0.08), I*** ^***2***^ ***= 0%***	16	1863	−0.09% (−3.10; 2.91), *I* ^*2*^ = 54%	20	2259	−2.19% (−6.63; 2.25), *I* ^*2*^ = 66%	Female	4	338	0.50% (−7.14; 8.14), *I* ^*2*^ = 47%
																	Male	4	461	2.69% (−2.44; 7.82), *I* ^*2*^ = 0%
	*ε4 vs. ε3*	20	4430	−0.003 (−0.03; 0.02), *I* ^*2*^ = 43%	16	2678	−0.02 (−0.06; 0.02), *I* ^*2*^ = 63%	5	815	***0.03 (0.005; 0.05), I*** ^***2***^ ***= 0%***	18	3305	−0.71% (−3.76; 2.35), *I* ^*2*^ = 96%	28	4644	***−10.08% (−15.30; −4.85), I*** ^***2***^ ***= 97%***	Female	4	437	−3.09% (−7.58; 1.39), *I* ^*2*^ = 4%
																	Male	4	564	***4.08% (0.80; 7.36), I*** ^***2***^ ***= 46%***
Non‐HDLC (mmol/L)	*ε4 vs. ε3*	NA	NA	NA	NA	NA	NA	NA	NA	NA	2	158	0.85% (−3.24; 4.94), *I* ^*2*^ = 0%	NA	NA	NA	NA	NA	NA	NA
Apo AI (mg/dL)	*ε2 vs. ε3*	4	295	−0.84 (−7.45; 5.77), *I* ^*2*^ = 0%	5	405	−0.54 (−8.15; 7.08), *I* ^*2*^ = 0%	NA	NA	NA	4	376	1.73% (−2.07; 5.53), *I* ^*2*^ = 0%	NA	NA	NA	NA	NA	NA	NA
	*ε4 vs. ε3*	3	350	−5.04 (−10.94; 0.85), *I* ^*2*^ = 0%	4	491	−4.18 (−10.16; 1.81), *I* ^*2*^ = 0%	NA	NA	NA	4	462	0.19% (−2.75; 3.14), *I* ^*2*^ = 38%	NA	NA	NA	NA	NA	NA	NA
Apo B (mg/dL)	*ε2 vs. ε3*	6	1282	***−12.97 (−16.10; −9.83), I*** ^***2***^ ***= 6%***	6	428	***−14.18 (−25.86; −2.50), I*** ^***2***^ ***= 70%***	NA	NA	NA	4	376	−3.50% (−11.62; 4.62), *I* ^*2*^ = 86%	NA	NA	NA	NA	NA	NA	NA
	*ε4 vs. ε3*	5	1512	2.51 (−0.28; 5.30), *I* ^*2*^ = 0%	5	517	−0.11 (−5.35; 5.14), *I* ^*2*^ = 0%	NA	NA	NA	4	462	1.86% (−3.05; 6.78), *I* ^*2*^ = 72%	NA	NA	NA	NA	NA	NA	NA
Apo E (mg/dL)	*ε2 vs. ε3*	3	1085	4.52 (−2.77; 11.81), *I* ^*2*^ = 92%	2	121	3.55 (−5.66; 12.76), *I* ^*2*^ = 97%	NA	NA	NA	2	202	***−18.49% (−26.38; −10.60), I*** ^***2***^ ***= 3%***	NA	NA	NA	NA	NA	NA	NA
	*ε4 vs. ε3*	2	1259	***−0.29 (−0.44; −0.13), I*** ^**2**^ ***= 0%***	NA	NA	NA	NA	NA	NA	2	235	−2.42% (−15.16; 10.32), *I* ^*2*^ = 51%	NA	NA	NA	NA	NA	NA	NA

*Note*: Statistically significant estimates (those with 95% CIs that do not cross 0) are italicized and bolded.

^a^ For a meta‐analysis of at least 10 studies with evidence of publication bias based on the linear regression test of funnel plot asymmetry *p* value < 0.1.

^b^ Pooled estimates are presented as mean differences (95% CI), heterogeneity.

Abbreviations: Apo AI, apolipoprotein AI; Apo B, apolipoprotein B; Apo E, apolipoprotein E; CI, confidence intervals; FH, familial hypercholesterolaemia; HDLC, high‐density lipoprotein cholesterol; *I* ^2^, I‐squared; a measure of heterogeneity; LDLC, low‐density lipoprotein cholesterol; Lp(a), lipoprotein(a); *n*, number of studies; *N*, number of participants; NA, not applicable/available; TC, total cholesterol; TG, total triglycerides.

**FIGURE 4:**
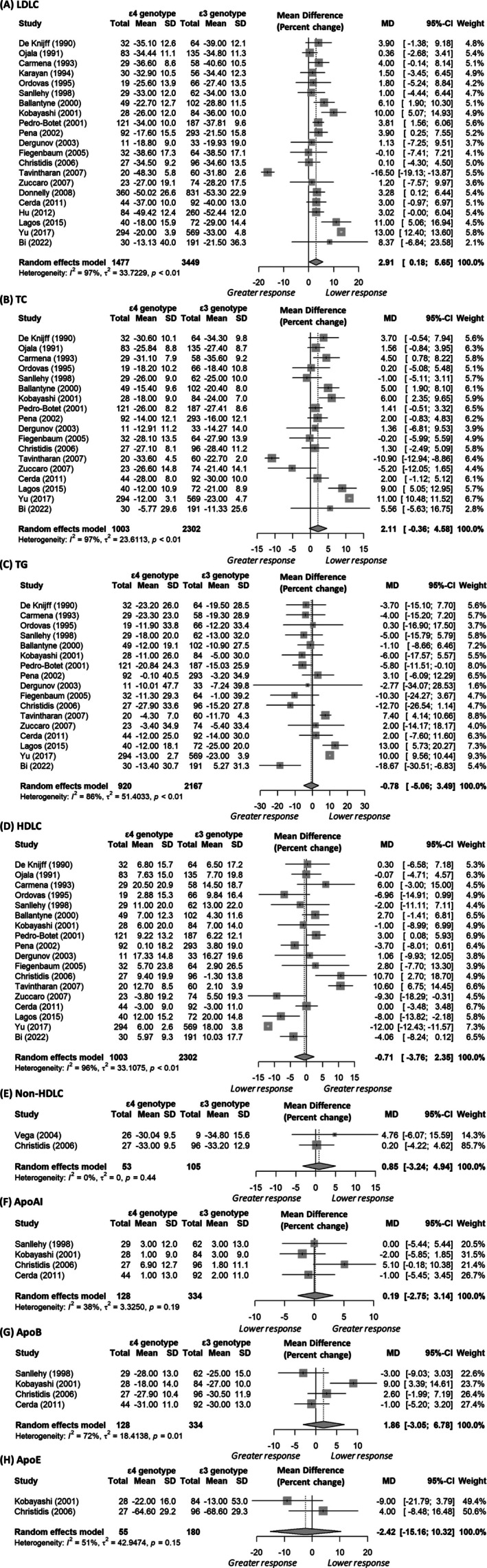
Forest plots comparing apolipoprotein *ε4* carriers with *ε3* carriers, excluding individuals with the *ε2ε4* genotype. For all biomarkers except HDLC and ApoAI, values greater than zero indicate a lower response to statin treatment in *ε4* carriers compared to *ε3* carriers (controls). Abbreviations: ApoAI, apolipoprotein AI; ApoB, apolipoprotein B; ApoE, apolipoprotein E; HDLC, high‐density lipoprotein cholesterol; LDLC, low‐density lipoprotein cholesterol; TC, Total cholesterol; TG, Total triglycerides.

In contrast, LDLC levels for *ε4 vs. ε3* carriers were similar both before (26 studies, 6275 participants; mean difference: 0.01 mmol/L, 95% CI: −0.09 to 0.11, *I* ^2^ = 68%) and after statin treatment (21 studies, 4178 participants; mean difference: 0.08 mmol/L, 95% CI: −0.10 to 0.25, *I* ^2^ = 96%). *ε4* carriers showed a less pronounced percentage decrease in LDLC compared to *ε3* carriers (21 studies, 4926 participants; mean difference: 2.91%, 95% CI: 0.18–5.65%, Figure 4A). However, heterogeneity was high (*I* ^2^ = 97%), and there was evidence of publication bias (linear regression test of funnel plot asymmetry *P* = .002; Figure S2). Sex‐stratified analysis explained some of the heterogeneity, with results becoming significant in both females (6 studies, 1053 participants; mean difference: 3.40%, 95% CI: 0.50% to 6.29%, *I* ^2^ = 44%) and males (6 studies, 1235 participants; mean difference: 3.91%, 95% CI: 1.55% to 6.27%, *I* ^2^ = 22%). A subgroup analysis of only Familial Hypercholesterolemia participants (2 studies, 530 participants; mean difference: 2.51%, 95% CI: 0.24% to 4.77%, *I* ^2^ = 0%) also accounted for the heterogeneity, with the results remaining significant. A trim‐and‐fill analysis estimated 11 missing studies (32 studies in total, 6326 participants), suggesting that these missing trials would further reduce the LDLC response to statins in *ε4* carriers to 10.04% (95% CI: 6.04% to 14.04%, *I* ^2^ = 98%; Figure S2). Consistent with the percentage change, *ε4* carriers had a smaller net reduction in LDLC levels by 0.30 mmol/L (95% CI: 0.06 to 0.53, *I* ^2^ = 74%; 5 studies, 1032 participants; Table 1 and Figure S3).

When individuals with the *ε2ε4* genotype were included in the analysis and categorized under *ε2* carriers (Table S9), *ε3* carriers (Table S10) or *ε4* carriers (Table S11), the results remained consistent with the above results that excluded the *ε2ε4* genotype category. Because the analyses, both including and excluding the *ε2ε4* genotype, showed similar patterns across biomarkers, the subsequent results will focus on analyses that exclude the *ε2ε4* genotype. Additionally, the focus will be on the change in biomarkers. However, full results, including comparisons of *ε2 vs*. non‐*ε2* and *ε4 vs*. non‐*ε4* carriers, are available in Tables S8–S11.

#### 3.2.2 Total cholesterol (TC)

No significant associations were found between the percentage (16 studies, 1863 participants; mean difference: −2.73%, 95% CI: −5.62% to 0.16%, *I* ^2^ = 86%, Figure 3C) and net (4 studies, 628 participants; mean difference: −0.22 mmol/L, 95% CI −0.49 to 0.05, *I* ^2^ = 49%; Figure S1) changes in TC levels between the *ε2* and *ε3* carriers. However, in a sex‐stratified analysis, male *ε2* carriers showed a greater percentage reduction in TC levels compared to male *ε3* carriers (4 studies, 461 participants; mean difference: −6.27%, 95% CI: −9.12% to −3.41%, *I* ^2^ = 3%). For the *ε4 vs. ε3* comparison, a significant association was observed for the net (4 studies, 815 participants; mean difference: 0.20 mmol/L, 95% CI 0.05 to 0.34, *I* ^2^ = 38%; Figure S3) but not percentage change (18 studies, 3305 participants; mean difference: 2.11%, 95% CI: −0.36% to 4.58%, *I* ^2^ = 97%, Figure 4C). For the percentage change, there was some evidence of publication bias (linear regression test of funnel plot asymmetry *P* = .004; Figure S4). A trim‐and‐fill analysis estimated 10 missing studies (28 studies in total, 4583 participants), and when these were accounted for, it suggested that *ε4* carriers had a lower TC response to statins by 8.99% (95% CI: 5.08% to 12.90%, *I* ^2^ = 98%; Figure S4).

#### 3.2.3 Total triglycerides (TG)

Similar to total cholesterol, no significant associations were found between the percentage (15 studies, 1722 participants; mean difference: −4.95%, 95% CI: −11.93% to 2.04%, *I* ^2^ = 78%, Figure 3B) and net (5 studies, 661 participants; mean difference: −0.17 mmol/L, 95% CI −0.33 to 0.0003, *I* ^2^ = 0%; Figure S1) changes in TG levels between the *ε2* and *ε3* carriers, even after conducting a trim‐and‐fill analysis to address evidence of publication bias for the percentage change (Figure S5). Similarly, no significant associations were observed for the *ε4 vs. ε3* comparison (percentage change mean difference [17 studies, 3087 participants]: −0.78%, 95% CI: −5.06% to 3.49%, *I* ^*2*^ = 86%, Figure 4B; and net change mean difference [5 studies, 851 participants]: −0.04 mmol/L, 95% CI: −0.12 to 0.04, *I* ^2^ = 0%, Figure S3). However, there was some evidence of publication bias (linear regression test of funnel plot asymmetry *P* < .001 for the percentage change; Figure S6). A trim‐and‐fill analysis estimated nine missing studies, which, when accounted for, suggested that *ε4* carriers, compared to *ε3* carriers, had a lower TG response to statins by 8.24% (95% CI: 2.15% to 14.33%, *I* ^2^ = 88%; 26 studies, 4265 participants; Figure S6).

#### 3.2.4 High‐density lipoprotein cholesterol (HDLC)

Based on 16 studies (1863 participants), *ε2* carriers had a similar percentage change in HDLC levels compared to *ε3* carriers (mean difference: −0.09%, 95% CI: −3.10% to 2.91%, *I* ^2^ = 54%, Figure 3D), even after conducting a trim‐and‐fill analysis to address evidence of publication bias (Figure S7). However, the net increase in HDLC levels for *ε2* carriers was significantly higher by 0.04 mmol/L (95% CI: 0.004 to 0.08, *I* ^2^ = 0%; 5 studies, 628 participants; Figure S1). In a comparable way, *ε4* carriers had a similar percentage change in HDLC levels compared to *ε3* carriers (18 studies, 3305 participants; mean difference: −0.71%, 95% CI: −3.76% to 2.35%, *I* ^2^ = 96%, Figure 4D) but an unexpectedly higher increase in net HDLC levels (5 studies, 815 participants; mean difference: 0.03 mmol/L, 95% CI 0.005 to 0.05, *I* ^2^ = 0%). However, there was evidence of publication bias (linear regression test of funnel plot asymmetry *P* < .001 for the percentage change; Figure S8). A trim‐and‐fill analysis estimated 10 missing studies, which, when accounted for, suggested that *ε4* allele carriers had a lower statin response (smaller increase in HDLC levels) compared to *ε3* carriers (28 studies, 4644 participants; mean difference: −10.08%, 95% CI: −15.30% to −4.85%, *I* ^2^ = 97%; Figure S8). In contrast, male *ε4* carriers had a greater increase in HDLC levels than male *ε3* carriers, with a mean difference of 4.08% (95% CI: 0.80% to 7.36%, *I* ^*2*^ = 46%) from 4 studies (564 participants).

#### 3.2.5 Other biomarkers

Except for Apolipoprotein E (ApoE), where *ε2* carriers showed a significantly greater reduction in ApoE levels compared to *ε3* carriers (2 studies, 202 participants; mean difference: −18.49%, 95% CI: −26.38% to −10.60%, *I* ^2^ = 3%, Figure 3G), none of the remaining biomarkers listed in Table 1 showed significant percentage differences.

Additional analyses (Figure S9) included LDLC and rs7412 (CT/TT *vs*. CC, TT *vs*. CC, and CT *vs*. CC), total cholesterol and rs7412 (CT/TT *vs*. CC, and CT *vs*. CC), mortality and *ε4* carrier status, risk of lobar intracranial haemorrhage and *ε2ε4*/*ε4ε4* genotypes. Significant associations were found only for the following:

- LDLC and rs7412 (TT *vs*. CC) with a mean difference of −4.58 (95% CI: −7.86 to −1.30, *I* ^2^ = 0%) across 2 studies with 2701 participants.
- Risk of lobar intracranial haemorrhage and *ε2ε4* genotypes (compared to *ε3ε3*), with an odds ratio of 7.60 (95% CI: 4.91 to 11.77, *I* ^2^ = 0%) across 2 studies with 1237 participants.
- Risk of lobar intracranial haemorrhage and *ε4ε4* genotypes (compared to *ε3ε3*), with an odds ratio of 6.66 (95% CI: 2.52 to 17.61, *I* ^2^ = 0%) across 2 studies with 1238 participants.

### 3.3 ASReview performance

**FIGURE 5:**
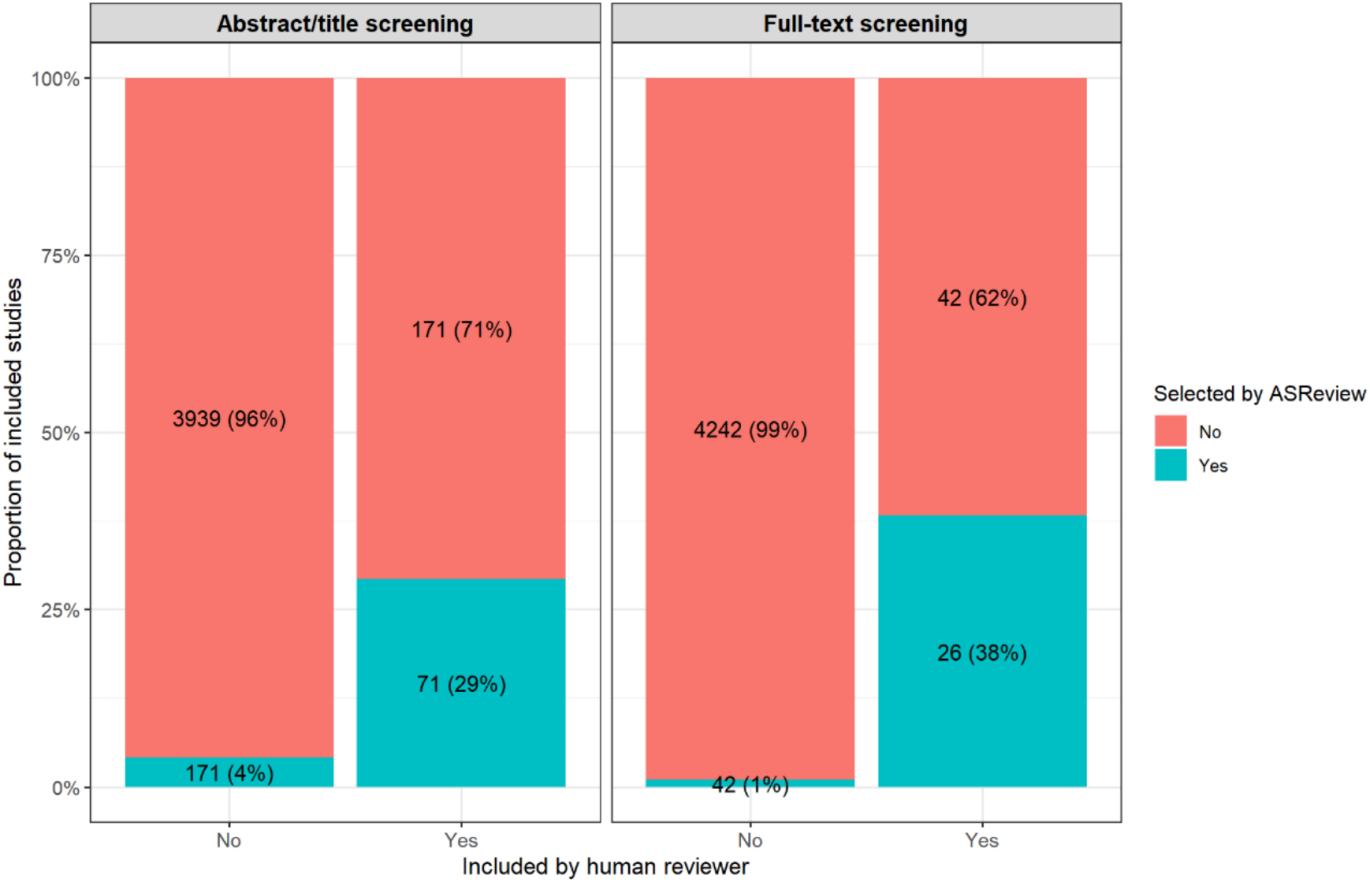
Proportion of articles selected by ASReview that were also included by human reviewers.

Figure 5 shows the proportion of articles selected by ASReview‐assisted screening that matched those included by human reviewers. Out of the 242 studies selected by the human reviewers during abstract/title screening (excluding six additional records identified through reference list searching; Figure 1), ASReview ranked 71 (29%) of these studies among its top 242 selections. This corresponds to a sensitivity of 29.3% (71 out of 242) and a specificity of 95.8% (3939 out of 4110), indicating that although ASReview accurately identified most studies not included by human reviewers, it was less successful in identifying those that were included. When the analysis was limited to the 68 studies selected by human reviewers during full‐text screening, ASReview ranked 26 (38%) of these studies among its top 68 selections, which yields a sensitivity of 38.2% (26 out of 68) and a specificity of 99.0% (4242 out of 4284). Details on the ASReview rankings and corresponding human screening results are shown in Table S12.

## 4 DISCUSSION

We have quantified the association between *APOE* genotype and responses to statins, focusing on the lipid biomarkers, low‐density lipoprotein cholesterol (LDLC), total cholesterol (TC), total triglycerides (TG) and high‐density lipoprotein cholesterol (HDLC). Compared to *ε3* carriers, *ε2* carriers showed a more pronounced response to statins with a greater reduction in LDLC (mean difference: −2.98%, 95% CI: −5.88% to −0.08%), but with similar reductions in TC (−2.73%, −5.62% to 0.16%), and TG (−4.95%, −11.93% to 2.04%), and a similar increase in HDLC (−0.09%, −3.10% to 2.91%). In contrast, after accounting for publication bias, *ε4* carriers, compared with *ε3* carriers experienced smaller reductions in LDLC (10.04%, 6.04% to 14.04%), TC (8.99%, 5.08% to 12.90%), and TG (8.24%, 2.15% to 14.33%), and a smaller increase in HDLC (−10.08%, −15.30% to −4.85%). Previous studies^8^, ^9^, ^13^, ^14^, ^90^, ^91^, ^92^ have shown some varying results, but in general, our findings are consistent with the overall trend of a differential response to statins between *ε2* and *ε4* carriers, when compared with *ε3* carriers. This is also consistent with the biological effects of the *ε2* and *ε4* isoforms in terms of binding to LDL receptors and subsequent downstream effects, including clearance.^6^, ^8^, ^9^

All *APOE* genotypes derive benefits from statin therapy: The mean percentage reductions in LDLC for *ε2*, *ε3* and *ε4* carriers were 36.0%, 34.1%, and 31.1%, respectively (Figures 3 and 4A), which is consistent with the reported mean differences (weighted) of −3.54% (*ε2 vs. ε3*) and 2.84% (*ε4 vs. ε3*, before trim‐fill analysis). We have focused on percentage changes because net changes do not account for the varying baseline levels across genotypes, and fewer studies reported net changes. Nonetheless, net changes were reported by some studies; for example, based on five studies, the mean difference in LDLC between *ε2* and *ε3* carriers was −0.14 mmol/L (95% CI: −0.28 to 0.003), whereas for *ε4 vs. ε3*, it was 0.30 mmol/L (95% CI: 0.06–0.53) (Table 1 and Figures S1 and S3). All *APOE* genotypes benefited from statin therapy, with the mean net reductions in LDLC levels for *ε2*, *ε3* and *ε4* carriers being −1.68, −1.52 and −1.23 mmol/L, respectively. It is important to note that these reductions are clinically significant. A prospective meta‐analysis involving 90 056 individuals across 14 randomized statin trials found that a 1 mmol/L reduction in LDLC was associated with a 21% reduction in the risk of major coronary events and a 19% reduction in coronary mortality over a 5‐year follow‐up period.^93^ Although *ε4* carriers tend to be more resistant to statin therapy, they may show greater percentage reductions in biomarker levels in response to treatment when their baseline levels are significantly higher (or lower for HDLC) than those of *ε2* or *ε3* carriers, who typically start with lower (or upper for HDLC) baseline levels.^12^ As shown in Table 1, baseline biomarker levels for *ε4* and *ε3* genotypes were similar prior to statin treatment, which indicates that the percentage changes observed were probably attributable to statin treatment rather than baseline levels. One potential explanation for the similar baseline levels is that prior non‐statin interventions, such as lipid‐lowering diets, may mask the effects of *APOE* genotypes on plasma lipid levels.^34^, ^38^

Heterogeneity was generally high, which is also consistent with previous meta‐analyses, and which may be due to factors such as differences in study design, participant characteristics, genotyping procedures, types and doses of statins, duration of treatment and limited statistical power.^13^, ^93^ In our study, due to limited data, we conducted subgroup analyses based on only sex and Familial Hypercholesterolemia. These analyses were able to account for some or most of the observed heterogeneity. During the sex‐stratified analyses, we noticed that males tended to show more pronounced genotype effects. For example, the LDLC analysis comparing *ε2* and *ε3* carriers showed significant effects for males (mean difference: −5.07%, 95% CI: −7.92% to −2.22%) but not females (mean difference: −2.84%, 95% CI −8.81% to 3.14%). These findings are consistent with previous studies, which suggested that these sex differences in response to statin treatment may be linked to variability in immune activation and hormone levels.^71^, ^94^

To evaluate if a machine learning framework could enhance the efficiency of the screening process, we used ASReview,^18^ with stopping criteria based on the number of records selected by human reviewers. Whereas ASReview demonstrated high specificity (95.8% during abstract/title screening and 99.0% during full‐text screening), its sensitivity was lower (29.3% for abstract/title screening and 38.2% for full‐text screening). This indicates that ASReview effectively identified most studies not included by human reviewers but was less effective at identifying those that were included. In related research, Tran et al.^95^ found that OpenAI's GPT‐3.5 Turbo achieved sensitivities ranging from 81.1% to 96.5% and specificities from 25.8% to 80.4% under the balanced rule, and sensitivities from 94.6% to 99.8% and specificities from 2.2% to 46.6% under the sensitive rule. Thus, GPT‐3.5 Turbo, like ASReview, has the potential to reduce the number of citations requiring human screening, although it may miss some citations at the full‐text level. As machine learning technology evolves, its performance will improve, and open‐source frameworks like ASReview offer opportunities for collaborative enhancements.

In addition to significant heterogeneity, another limitation of our review was the small number of studies (two or fewer) available for certain biomarkers, such as ApoE. Furthermore, the lack of standardized genetic reporting hindered comparability across studies. Only a minority reported full genotype distributions (*ε2ε2*, *ε2ε3*, *ε2ε4*, *ε3ε3*, *ε3ε4* and *ε4ε4*), whereas others provided only *ε2*, *ε3* or *ε4* carrier status. We were also unable to account for the specific types and doses of statins used in the included studies, nor could we make dose‐equivalence adjustments, which is another key limitation, as not all statins have equal potency. From a clinical perspective, the reduced effect on lipid levels seen in *ε4* carriers could potentially be counteracted by higher (tolerated) doses, but this could not be evaluated. We also planned to use the STROPs checklist,^19^ but we were unable to apply this tool due to a frequent lack of required information (more than half of the studies were published before 2010 when reporting guidelines specifying mandatory information were not yet available). Regarding publication bias, we assessed funnel‐plot asymmetry using the linear regression test, which is a commonly used approach. Although additional tests such as Egger's could have been applied, doing so would introduce issues related to multiplicity without clear added value. We also used trim‐and‐fill analysis to estimate the potential impact of missing studies; however, this method assumes that funnel‐plot asymmetry reflects publication bias, whereas asymmetry may arise from heterogeneity or small‐study effects. The adjusted estimates are also less precise. Therefore, trim‐and‐fill results should be interpreted cautiously. Finally, we did not have sufficient data to report on clinical outcomes such as mortality, but as stated above, a reduction of 1 mmol/L in LDLC is associated with a 19% decrease in the risk of coronary mortality over a 5‐year period.^93^ Interestingly, our meta‐analysis identified an increased risk of lobar intracranial haemorrhage in *ε4* carriers, with high odds ratios (6.66–7.60). This is not surprising given that *ε4* carriage increases the risk of cerebral amyloid angiopathy and spontaneous intracerebral haemorrhage.^96^

To address some of these limitations, specifically the inability to adjust for statin dose and type and the lack of clinical outcome data, we conducted additional analyses using large datasets (UK Biobank and the US All of Us Research Program) with linked electronic health records.^15^ In the UK Biobank, *ε3ε4* (HR 1.08, 95% CI 1.01–1.15) and *ε4ε4* (HR 1.54, 95% CI 1.33–1.78) carriers had higher all‐cause mortality risk; in All of Us, only *ε4ε4* carriers showed increased risk (HR 1.64, 95% CI 1.08–2.49). However, we were unable to demonstrate a statistically significant *APOE*–statin interaction, nor were major adverse cardiovascular events significantly associated with genotype, highlighting the need for larger studies, ideally randomized controlled trials with genotyping.

## 5 CONCLUSION

Our meta‐analysis shows that the *APOE* genotype influences the effectiveness of statin therapy. *ε2* carriers generally show more pronounced reductions in lipid levels, indicating greater responsiveness to treatment, whereas *ε4* carriers show a comparatively weaker response. These findings should be considered alongside other risk factors, as homozygous *ε2* carriers, in rare cases, may be predisposed to familial dysbetalipoproteinemia, which increases cardiovascular disease risk due to abnormal lipid metabolism.^4^, ^11^ Personalized treatment strategies that consider *APOE* genotype could optimize lipid management and reduce cardiovascular risk across different patient populations. Although the Clinical Pharmacogenetics Implementation Consortium considers current evidence insufficient to support clinical guidelines for *APOE*–statin interactions, it classifies the *APOE*–atorvastatin association as provisional with a Level 2B evidence rating (moderate evidence; https://cpicpgx.org/genes-drugs/). It is important to note that we are not advocating determination of *APOE* genotype prior to statin therapy largely because of the ethical implications, but where the *APOE* genotype is known (and this is likely to increase), it should be considered in terms of both dose and potency of the statin.

## CONFLICT OF INTEREST STATEMENT

M.P. currently receives partnership funding, paid to the University of Liverpool, for the MRC Medicines Development Fellowship Scheme (co‐funded by MRC and GSK, AZ, Optum and Hammersmith Medicines Research). He has developed an HLA genotyping panel with MC Diagnostics but does not benefit financially from this. He is part of the IMI Consortium ARDAT (www.ardat.org); none of these of funding sources have been used for the current research. I.G.A. is currently employed by Novartis Pharmaceuticals UK Ltd, with employment commencing after all work described in this paper was completed. All other authors declared no competing interests for this work.

## Supporting information


**Figure S1:**Forest plots comparing Apolipoprotein *ε2* carriers with *ε3* carriers, excluding individuals with the *ε2ε4* genotype. All biomarkers are in mmol/L. For all biomarkers except HDLC, values greater than zero indicate a lower response to statin treatment in *ε2* carriers compared to *ε3* carriers (controls). Abbreviations: HDLC = High‐Density Lipoprotein Cholesterol, LDLC = Low‐Density Lipoprotein Cholesterol, TC = Total Cholesterol, TG = Total Triglycerides.
Figure S2: Funnel plot (Panel A) and Trim and fill analysis (Panel B) for the comparison between Low‐Density Lipoprotein Cholesterol and Apolipoprotein *ε4* carriers with *ε3* carriers, excluding individuals with the *ε2ε4* genotype. The p‐value for the linear regression test of funnel plot asymmetry is displayed at the top of the figure.
Figure S3: Forest plots comparing Apolipoprotein *ε4* carriers with *ε3* carriers, excluding individuals with the *ε2ε4* genotype. All biomarkers are in mmol/L. For all biomarkers except HDLC, values greater than zero indicate a lower response to statin treatment in *ε4* carriers compared to *ε3* carriers (controls). Abbreviations: HDLC = High‐Density Lipoprotein Cholesterol, LDLC = Low‐Density Lipoprotein Cholesterol, TC = Total Cholesterol, TG = Total Triglycerides.
Figure S4: Funnel plot (Panel A) and Trim and fill analysis (Panel B) for the comparison between Total Cholesterol and Apolipoprotein *ε4* carriers with *ε3* carriers, excluding individuals with the *ε2ε4* genotype. The p‐value for the linear regression test of funnel plot asymmetry is displayed at the top of the figure.
Figure S5: Funnel plot (Panel A) and Trim and fill analysis (Panel B) for the comparison between Total Triglycerides and Apolipoprotein *ε2* carriers with *ε3* carriers, excluding individuals with the *ε2ε4* genotype. The p‐value for the linear regression test of funnel plot asymmetry is displayed at the top of the figure.
Figure S6: Funnel plot (Panel A) and Trim and fill analysis (Panel B) for the comparison between Total Triglycerides and Apolipoprotein *ε4* carriers with *ε3* carriers, excluding individuals with the *ε2ε4* genotype. The p‐value for the linear regression test of funnel plot asymmetry is displayed at the top of the figure.
Figure S7: Funnel plot (Panel A) and Trim and fill analysis (Panel B) for the comparison between High‐Density Lipoprotein Cholesterol and Apolipoprotein *ε2* carriers with *ε3* carriers, excluding individuals with the *ε2ε4* genotype. The p‐value for the linear regression test of funnel plot asymmetry is displayed at the top of the figure.
Figure S8: Funnel plot (Panel A) and Trim and fill analysis (Panel B) for the comparison between High‐Density Lipoprotein Cholesterol and Apolipoprotein *ε4* carriers with *ε3* carriers, excluding individuals with the *ε2ε4* genotype. The p‐value for the linear regression test of funnel plot asymmetry is displayed at the top of the figure.
Figure S9: Forest plots for additional biomarkers. The reference genotype in panels G and H is *ε3ε3*.

**Table S1:** Preferred Reporting Items for Systematic Reviews and Meta‐Analyses (PRISMA) and Meta‐analysis Of Observational Studies in Epidemiology (MOOSE) Statements
Table S2: Search strategy (9th May 2024)
Table S3: Formulae for combining means and standard deviations.
Table S4: Characteristics of the studies included in the systematic review
Table S5: Extracted results for the ratio outcomes
Table S6: Extracted results for the binary outcomes
Table S7: Extracted results for the continuous outcomes
Table S8: Meta‐analysis results with the ε2ε4 genotype category excluded
Table S9: Meta‐analysis results with the ε2ε4 genotype category included under ε2 carriers
Table S10: Meta‐analysis results with the ε2ε4 genotype category included under ε3 carriers
Table S11: Meta‐analysis results with the ε2ε4 genotype category included under ε4 carriers
Table S12: ASReview rankings and corresponding human screening results

## Data Availability

The data underlying this article are available in the article and in its online supplementary material.
