# Supplementary material for: APOE genotype and the effect of statins on lipid outcomes: A meta‐analysis
Source: Br J Clin Pharmacol. 2026 Feb 17;92(5):1268–86. doi: 10.1002/bcp.70493 (PMC13122289; doi:10.1002/bcp.70493)
Supplement: Supplementary file 1 — Figure S1:Forest plots comparing Apolipoprotein ε2 carriers with ε3 carriers, excluding individuals with the ε2ε4 genotype. All biomarkers are in mmol/L. For all biomarkers except HDLC, values greater than zero indicate a lower response to statin treatment in ε2 carriers compared to ε3 carriers (controls). Abbreviations: HDLC = High‐Density Lipoprotein Cholesterol, LDLC = Low‐Density Lipoprotein Cholesterol, TC = Total Cholesterol, TG = Total Triglycerides. Figure S2: Funnel plot (Panel A) and Trim and fill analysis (Panel B) for the comparison between Low‐Density Lipoprotein Cholesterol and Apolipoprotein ε4 carriers with ε3 carriers, excluding individuals with the ε2ε4 genotype. The p‐value for the linear regression test of funnel plot asymmetry is displayed at the top of the figure. Figure S3: Forest plots comparing Apolipoprotein ε4 carriers with ε3 carriers, excluding individuals with the ε2ε4 genotype. All biomarkers are in mmol/L. For all biomarkers except HDLC, values greater than zero indicate a lower response to statin treatment in ε4 carriers compared to ε3 carriers (controls). Abbreviations: HDLC = High‐Density Lipoprotein Cholesterol, LDLC = Low‐Density Lipoprotein Cholesterol, TC = Total Cholesterol, TG = Total Triglycerides. Figure S4: Funnel plot (Panel A) and Trim and fill analysis (Panel B) for the comparison between Total Cholesterol and Apolipoprotein ε4 carriers with ε3 carriers, excluding individuals with the ε2ε4 genotype. The p‐value for the linear regression test of funnel plot asymmetry is displayed at the top of the figure. Figure S5: Funnel plot (Panel A) and Trim and fill analysis (Panel B) for the comparison between Total Triglycerides and Apolipoprotein ε2 carriers with ε3 carriers, excluding individuals with the ε2ε4 genotype. The p‐value for the linear regression test of funnel plot asymmetry is displayed at the top of the figure. Figure S6: Funnel plot (Panel A) and Trim and fill analysis (Panel B) for the comparison between Total T [file BCP-92-1268-s002.docx]

**
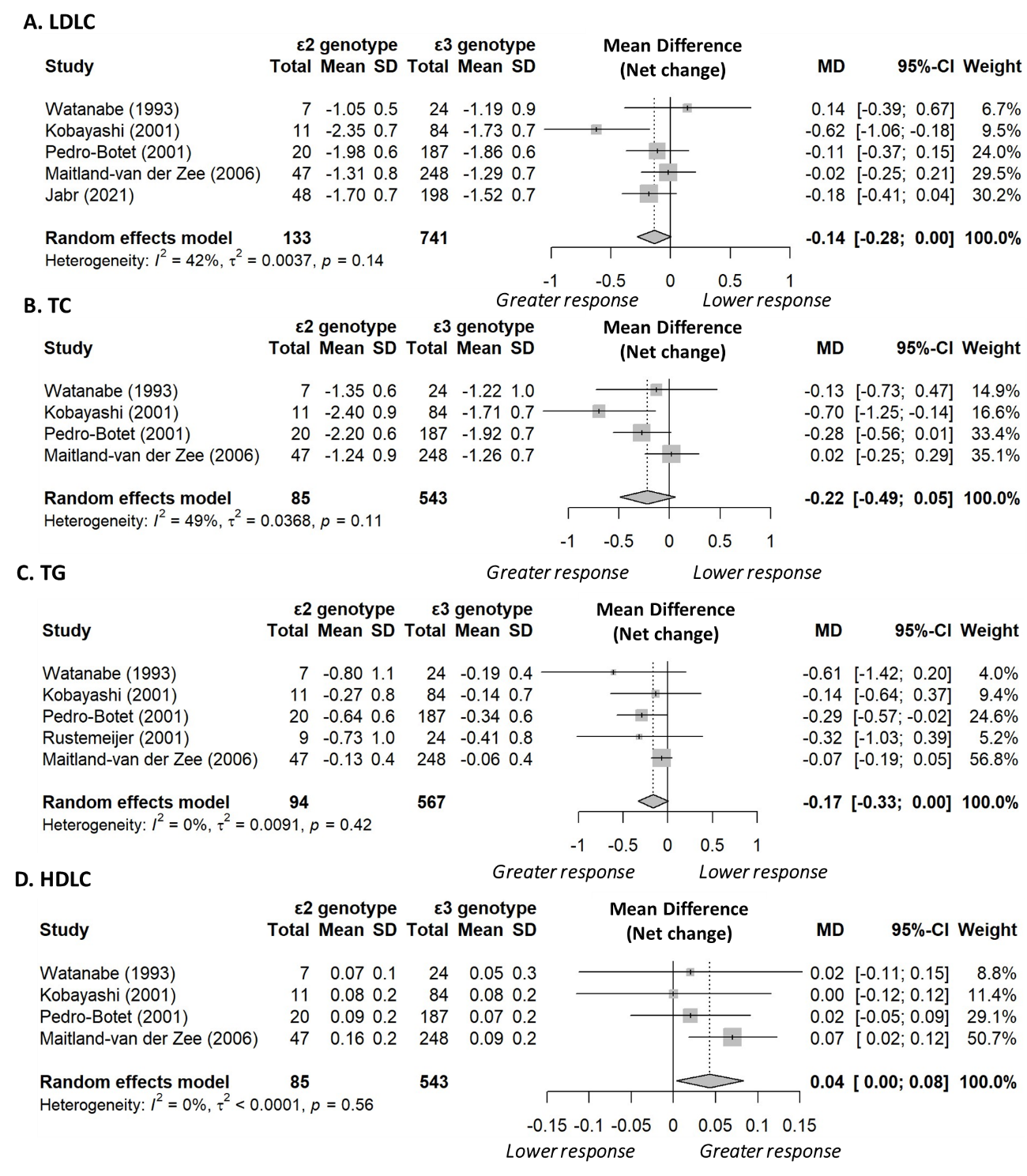
**

**Figure S1. Forest plots comparing Apolipoprotein** ***ε2* carriers with *ε3* carriers, excluding individuals with the *ε2ε4* genotype.** All biomarkers are in mmol/L. For all biomarkers except HDLC, values greater than zero indicate a lower response to statin treatment in *ε2* carriers compared to *ε3* carriers (controls). Abbreviations: HDLC = High-Density Lipoprotein Cholesterol, LDLC = Low-Density Lipoprotein Cholesterol, TC = Total Cholesterol, TG = Total Triglycerides.

**A.**

**
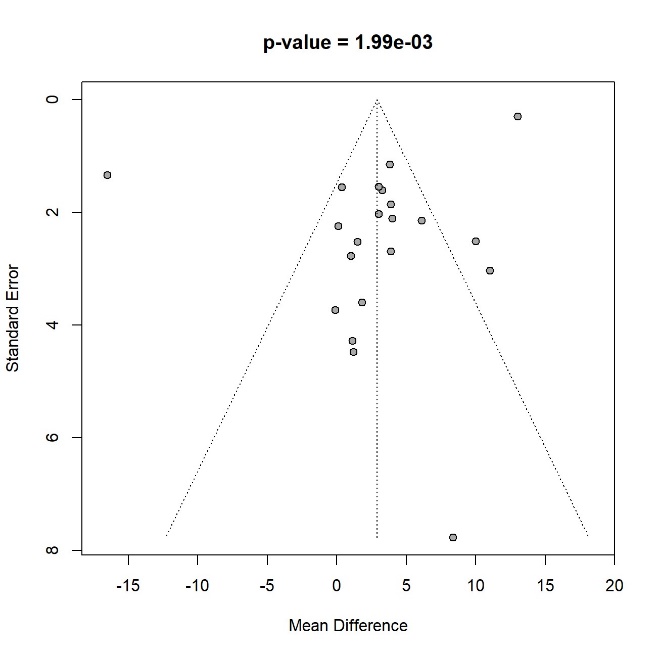
**

**Mean Difference (Percent change)**

**(Percentage**

***ε4*  *ε3***

**B.**

**
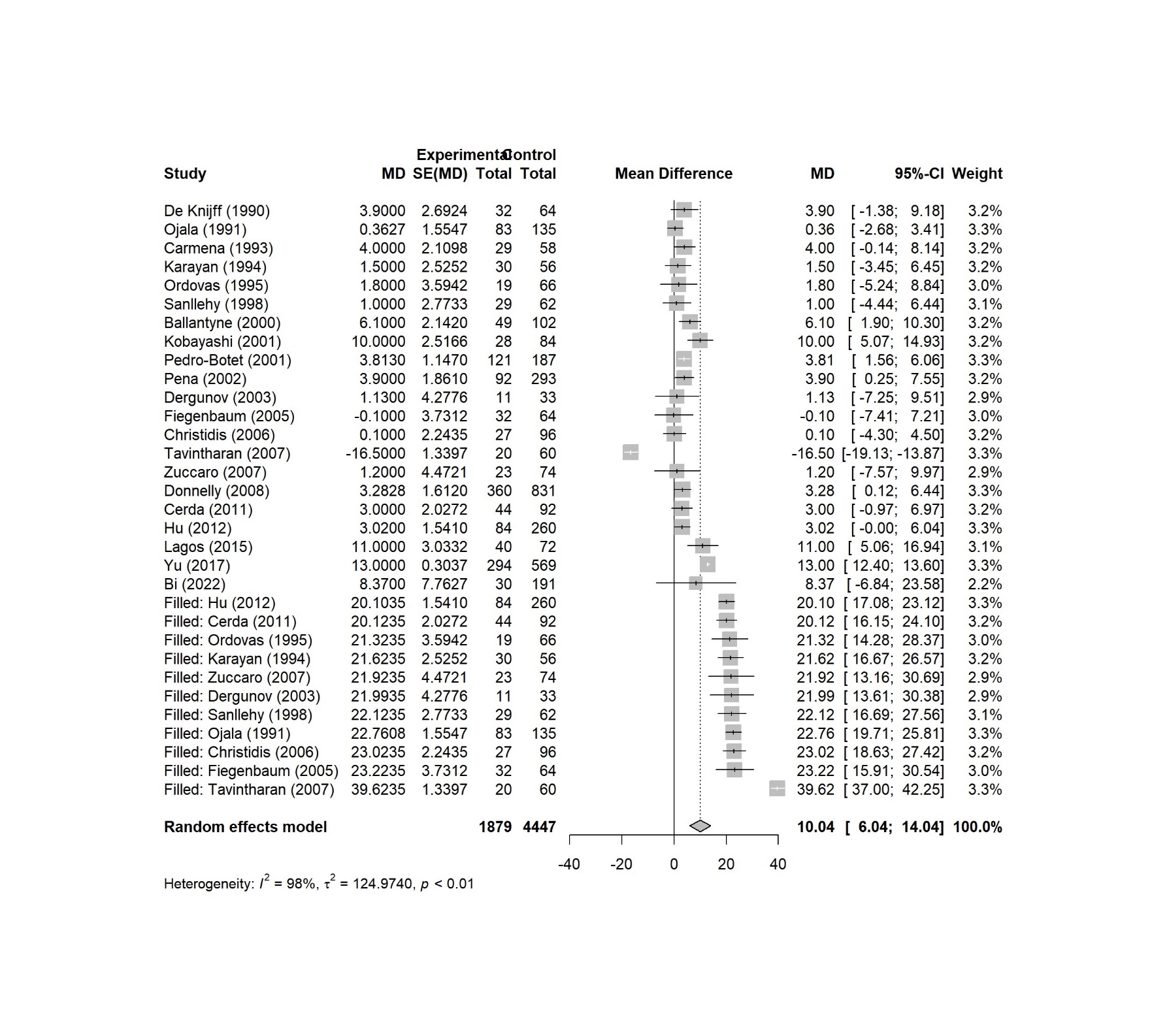
**

***Greater response Lower response***

**Figure S2. Funnel plot (Panel A) and Trim and fill analysis (Panel B) for the comparison between Low-Density Lipoprotein Cholesterol and Apolipoprotein *ε4* carriers with *ε3* carriers, excluding individuals with the *ε2ε4* genotype.** The p-value for the linear regression test of funnel plot asymmetry is displayed at the top of the figure.

**
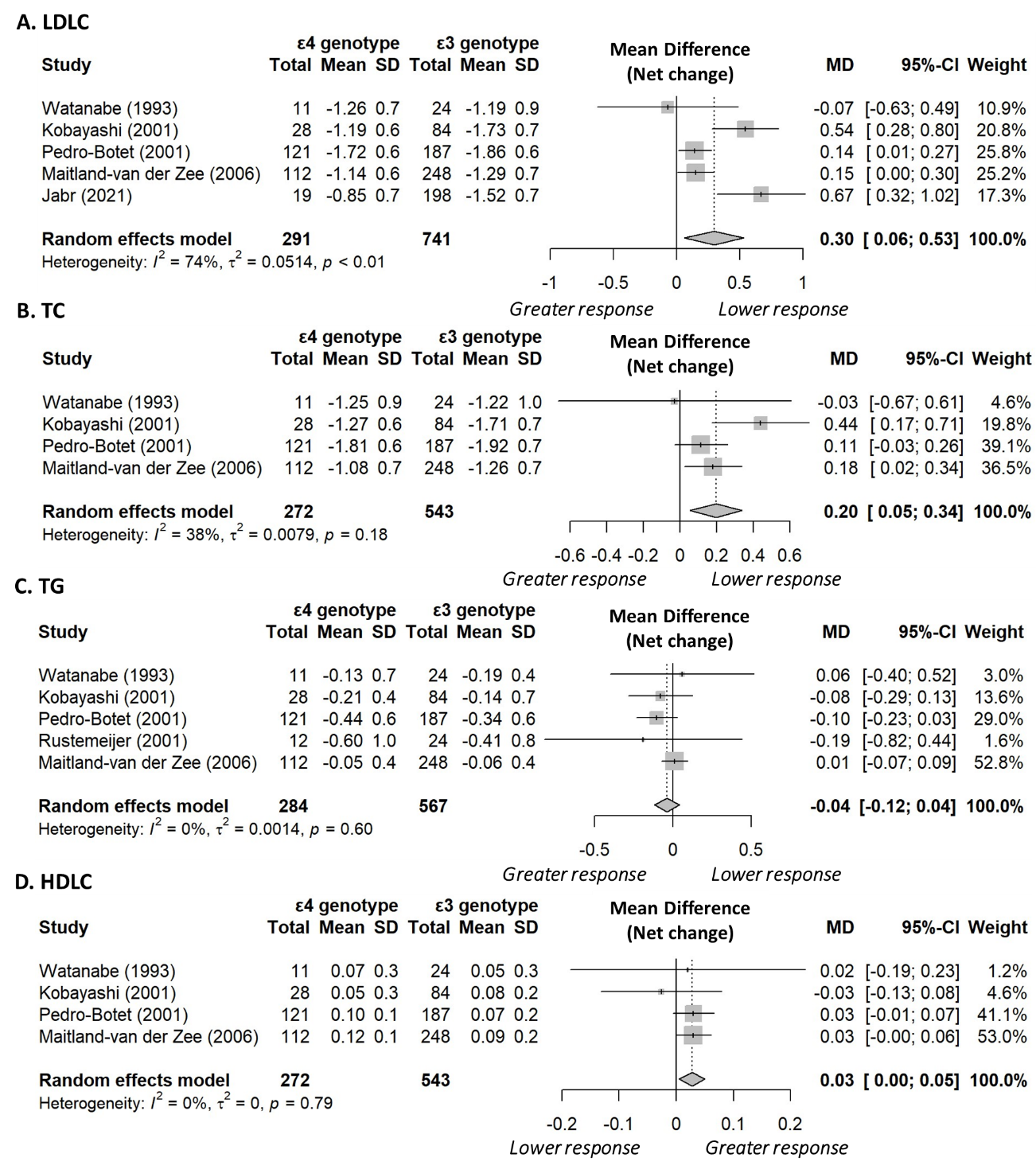
**

**Figure S3. Forest plots comparing Apolipoprotein *ε4* carriers with *ε3* carriers, excluding individuals with the *ε2ε4* genotype.** All biomarkers are in mmol/L. For all biomarkers except HDLC, values greater than zero indicate a lower response to statin treatment in *ε4* carriers compared to *ε3* carriers (controls). Abbreviations: HDLC = High-Density Lipoprotein Cholesterol, LDLC = Low-Density Lipoprotein Cholesterol, TC = Total Cholesterol, TG = Total Triglycerides.

**A.**

**
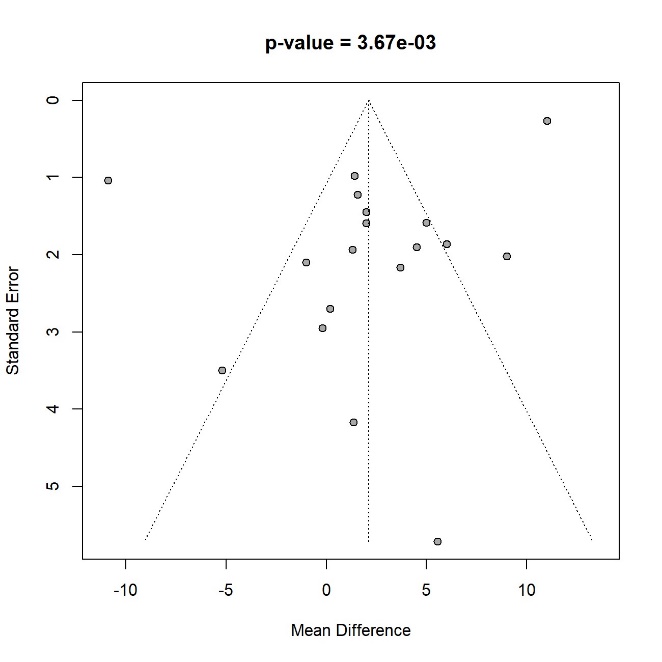
**

**Mean Difference (Percent change)**

**(Percentage**

***ε4*  *ε3***

**B.**

**
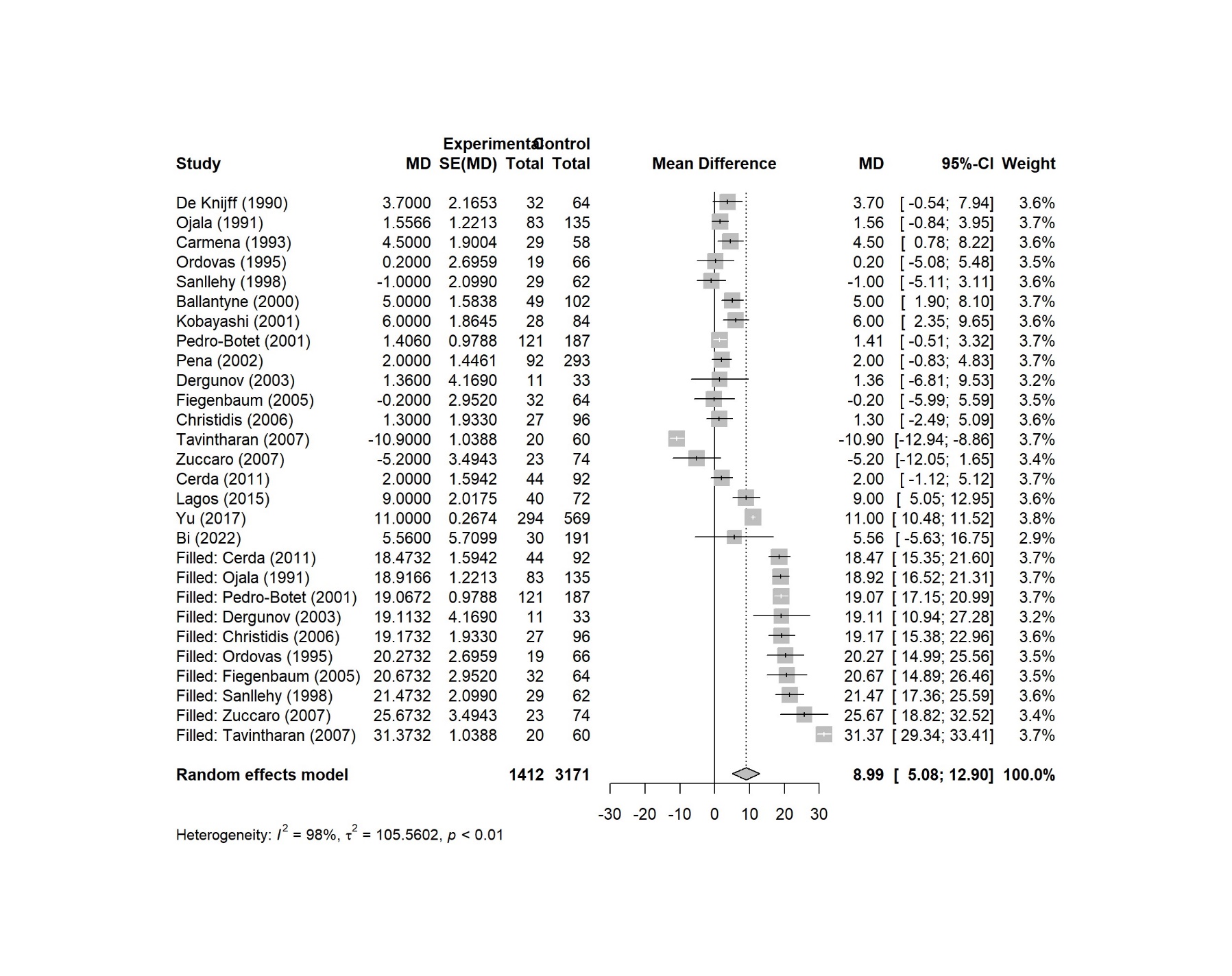
**

***Greater response Lower response***

**Figure S4. Funnel plot (Panel A) and Trim and fill analysis (Panel B) for the comparison between Total Cholesterol and Apolipoprotein *ε4* carriers with *ε3* carriers, excluding individuals with the *ε2ε4* genotype.** The p-value for the linear regression test of funnel plot asymmetry is displayed at the top of the figure.

**A.**

**
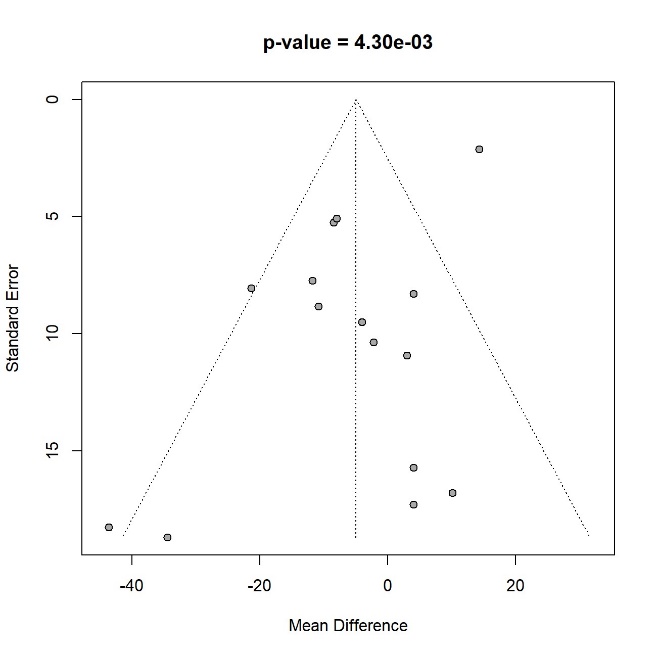
**

***ε2*  *ε3***

***Greater response Lower response***

**Mean Difference (Percent change)**

**(Percentage**

**B.**

**
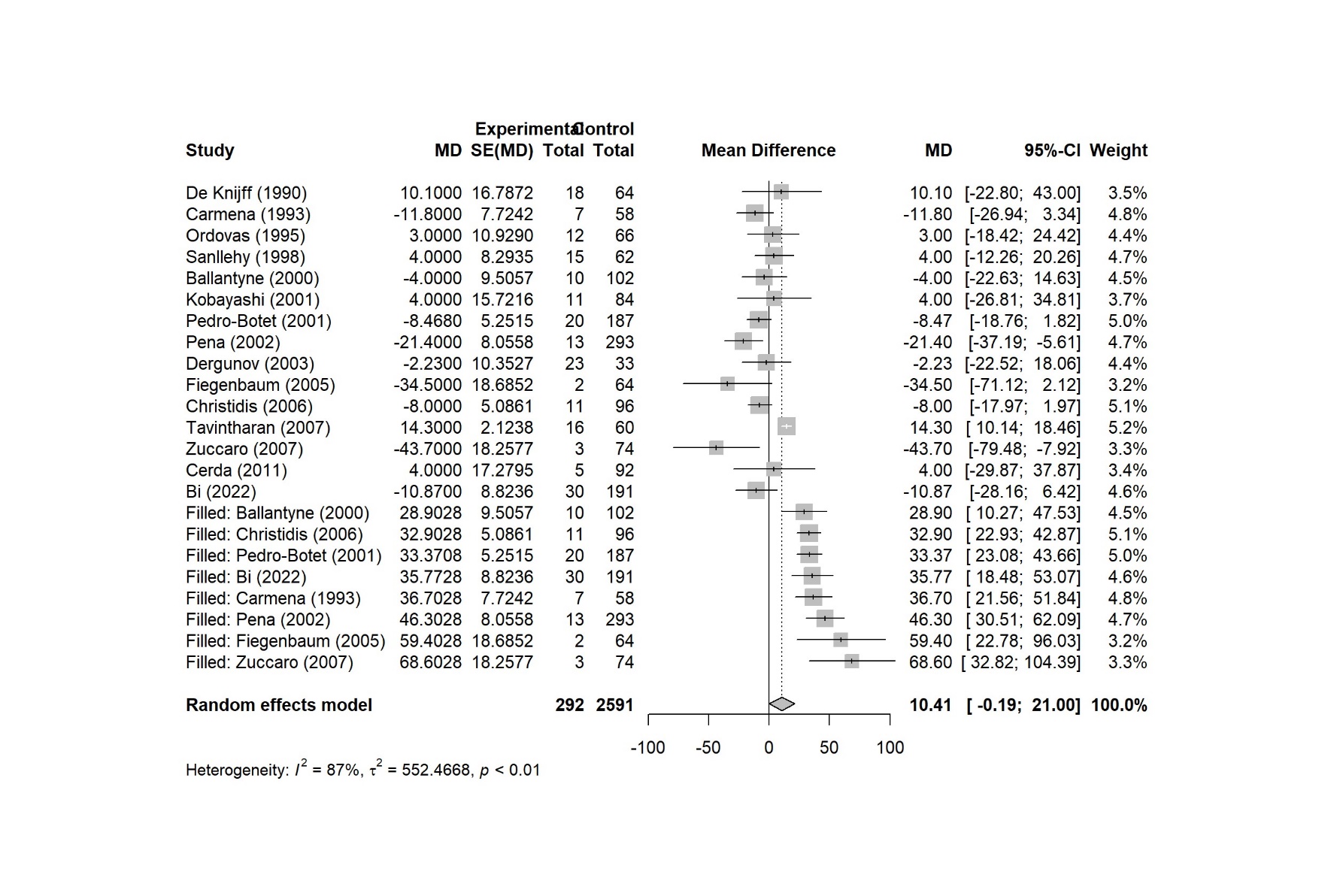
**

**Figure S5. Funnel plot (Panel A) and Trim and fill analysis (Panel B) for the comparison between Total Triglycerides and Apolipoprotein *ε2* carriers with *ε3* carriers, excluding individuals with the *ε2ε4* genotype.** The p-value for the linear regression test of funnel plot asymmetry is displayed at the top of the figure.

**A.**


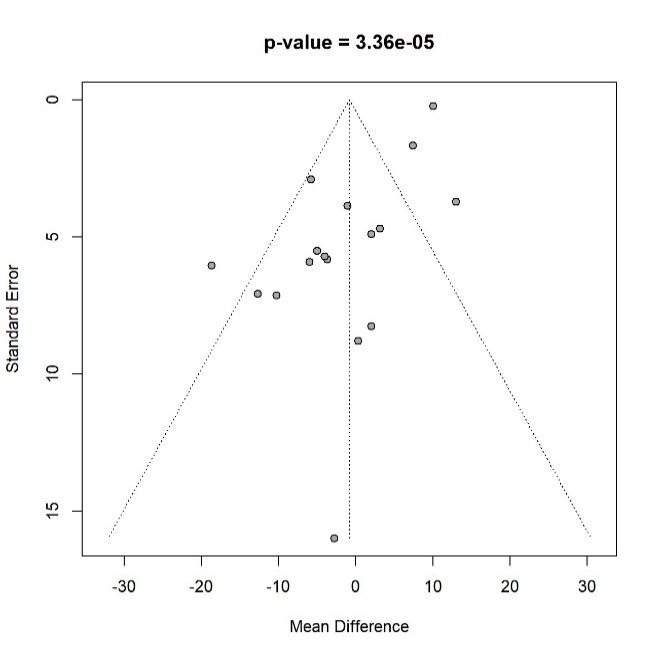


***ε4*  *ε3***

***Greater response Lower response***

**Mean Difference (Percent change)**

**(Percentage**

**B.**

**
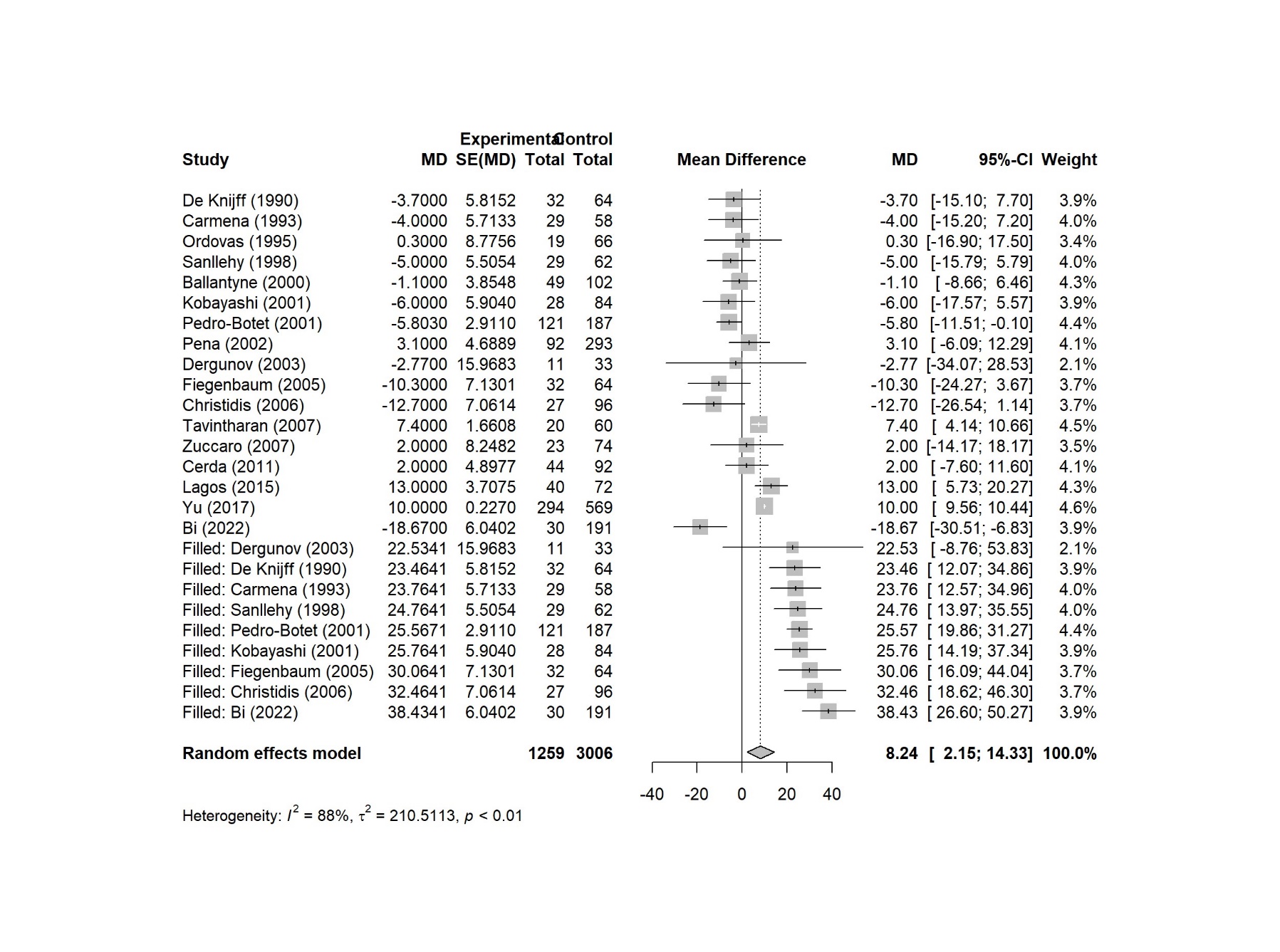
**

**Figure S6. Funnel plot (Panel A) and Trim and fill analysis (Panel B) for the comparison between Total Triglycerides and Apolipoprotein *ε4* carriers with *ε3* carriers, excluding individuals with the *ε2ε4* genotype.** The p-value for the linear regression test of funnel plot asymmetry is displayed at the top of the figure.

**A.**

**
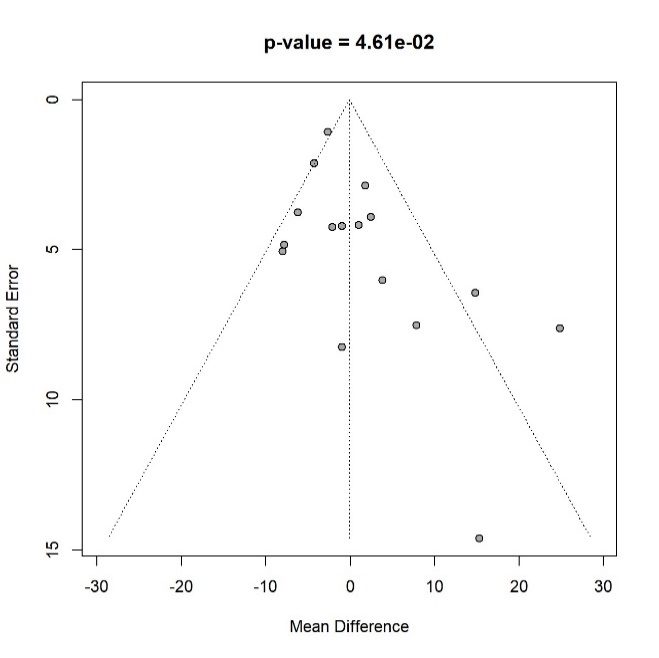
**

***ε2*  *ε3***

***Lower response Greater response***

**Mean Difference (Percent change)**

**(Percentage**

**B.**

**
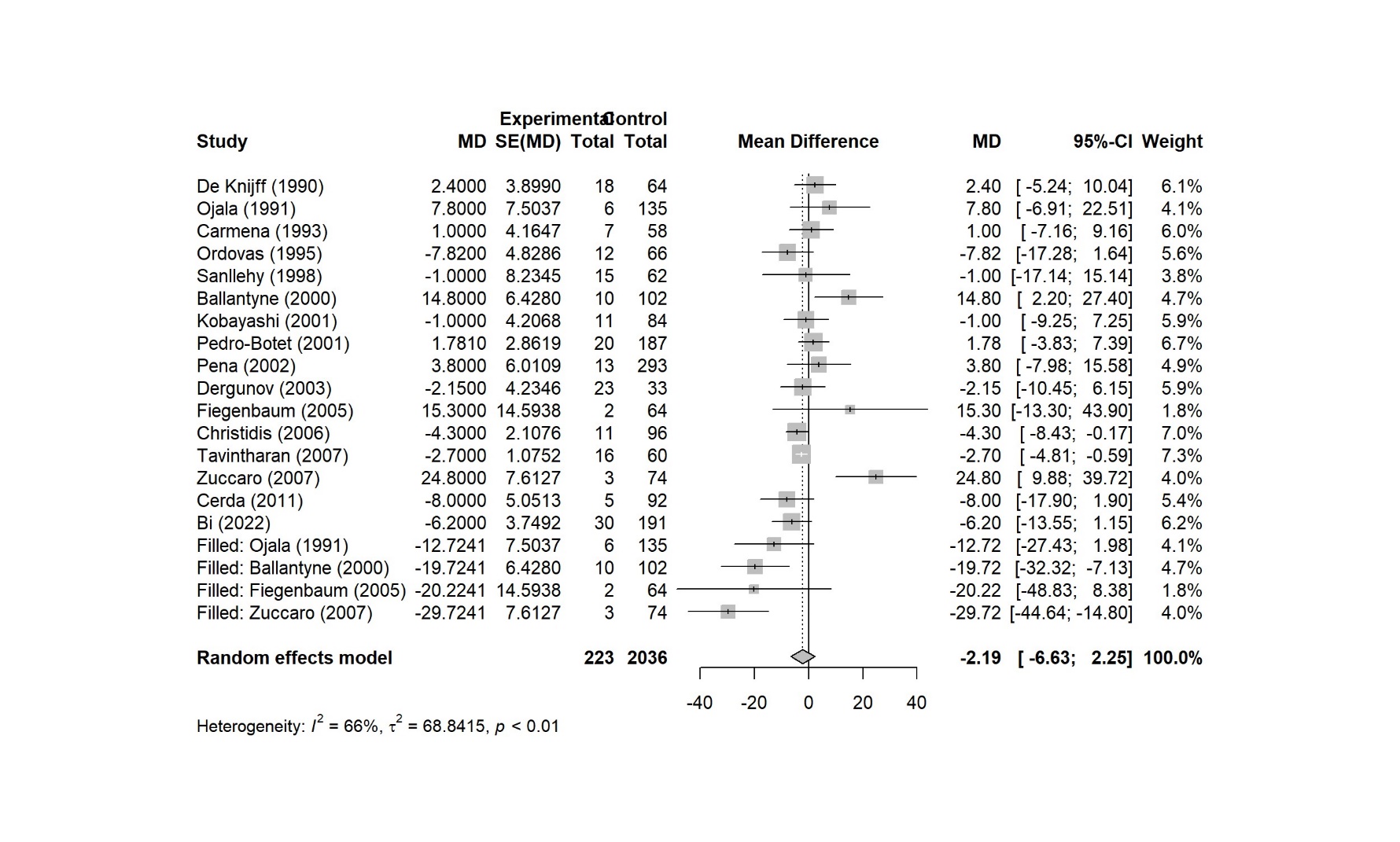
**

**Figure S7. Funnel plot (Panel A) and Trim and fill analysis (Panel B) for the comparison between High-Density Lipoprotein Cholesterol and Apolipoprotein *ε2* carriers with *ε3* carriers, excluding individuals with the *ε2ε4* genotype.** The p-value for the linear regression test of funnel plot asymmetry is displayed at the top of the figure.

**A.**

**
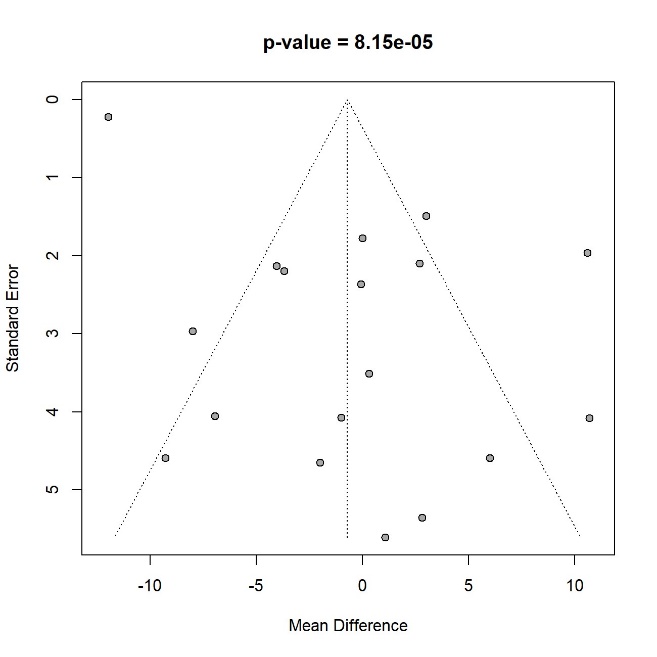
**

***ε4*  *ε3***

***Lower response Greater response***

**Mean Difference (Percent change)**

**(Percentage**

**B.**

**
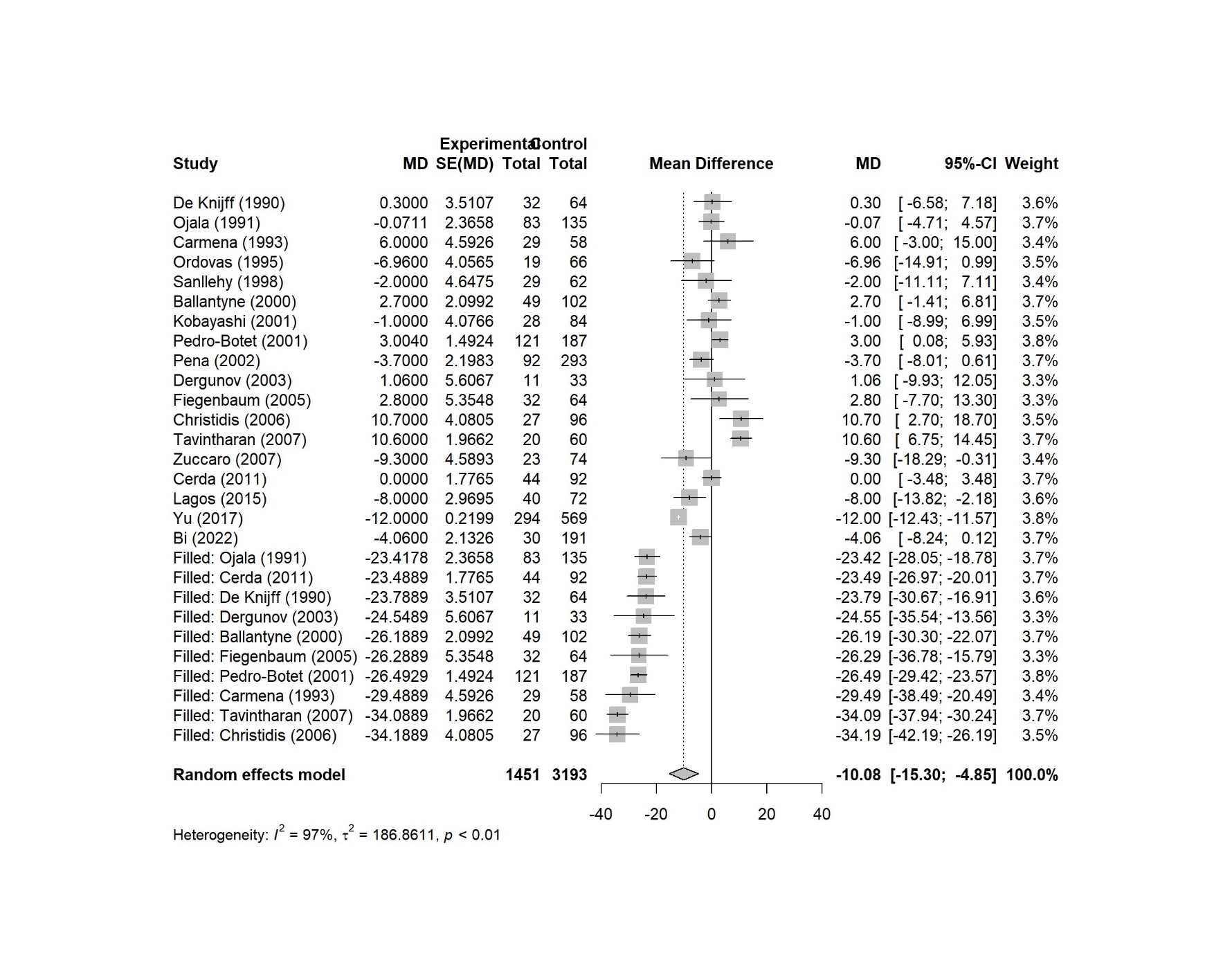
**

**Figure S8. Funnel plot (Panel A) and Trim and fill analysis (Panel B) for the comparison between High-Density Lipoprotein Cholesterol and Apolipoprotein *ε4* carriers with *ε3* carriers, excluding individuals with the *ε2ε4* genotype.** The p-value for the linear regression test of funnel plot asymmetry is displayed at the top of the figure.

**
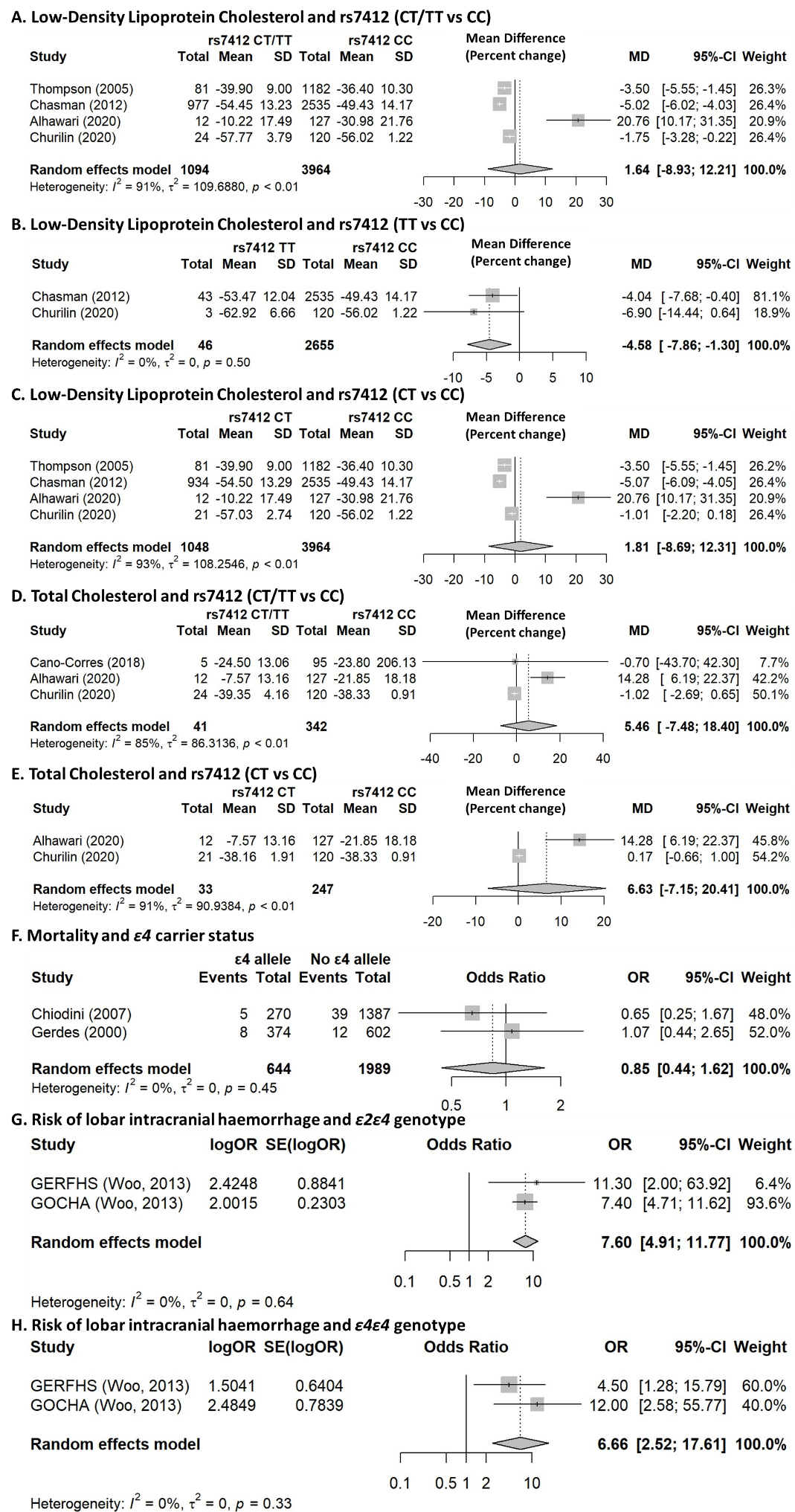
**

**Figure S9. Forest plots for additional biomarkers.** The reference genotype in panels G and H is *ε3ε3*.
